# Well-being of health workers providing maternal and newborn care: A qualitative evidence synthesis

**DOI:** 10.1371/journal.pgph.0005522

**Published:** 2026-02-11

**Authors:** Alya Hazfiarini, Martha Vazquez Corona, Nicole Minckas, Caroline S. E. Homer, Tari Turner, Rana Islamiah Zahroh, Patience A. Afulani, Giorgio Cometto, Steve McDonald, Charlotte E. Warren, Özge Tunçalp, Anayda Portela, Meghan A. Bohren

**Affiliations:** 1 Gender and Women’s Health Unit, Nossal Institute for Global Health, School of Population and Global Health, University of Melbourne, Carlton, Victoria, Australia; 2 Women, Children and Adolescent Health Program, Burnet Institute, Melbourne, Victoria, Australia; 3 School of Public Health and Preventive Medicine, Monash University, Melbourne, Victoria, Australia; 4 Department of Obstetrics Gynecology and Reproductive Sciences, University of California San Francisco, San Francisco, California, United States of America; 5 Heath Workforce, World Health Organization, Geneva, Switzerland; 6 Population Council Inc, New York, NY, United States of America; 7 Institute of Tropical Medicine Antwerp, Antwerp, Belgium; 8 Department of Sexual, Reproductive, Maternal, Child and Adolescent Health and Ageing, World Health Organization, Geneva, Switzerland; Jhpiego, UNITED STATES OF AMERICA

## Abstract

Health workers providing maternal and newborn care can experience burnout and emotional distress, which harms their well-being and, consequently, their ability to deliver respectful care. The World Health Organization (WHO) *Compendium on respectful maternal and newborn care* identifies health workers’ well-being as a critical area for intervention, but no specific definition or domains of well-being exist for this group. We conducted a qualitative evidence synthesis to synthesise key domains of well-being for health workers providing maternal and newborn care, by exploring health workers’ perceptions and experiences about their well-being. We searched MEDLINE, CINAHL and MIDIRS from 2010 to 20 August 2025 to identify studies reporting health workers’ perceptions and experiences of well-being while providing routine maternal and newborn care in any context. Using maximum variation purposive sampling, we sampled papers for data extraction. Thematic synthesis was employed for analysis, with GRADE-CERQual assessing confidence in findings. A mixed inductive-deductive approach informed the development of well-being domains. Potential implementation strategies to improve well-being were developed using Theoretical Domains Framework (TDF); Capability, Opportunity, and Motivation Model of Behaviour (COM-B); and Behaviour Change Wheel (BCW). Consultations with health workers validated findings and implementation strategies. We analysed 51 papers from five WHO regions, except the South-East Asia Region, with 61% from high-income and 39% from low-middle-income countries. We identified 18 findings on factors influencing health workers’ well-being. Eight key domains of well-being identified: 1) physical health and access to care and support, 2) emotional and psychological health and access to care and support, 3) fair, safe, and supportive environment, 4) fair and equitable remuneration, 5) adequate housing and a safe community, 6) strong professional identity, 7) positive relationship with women and their families, and 8) work and personal life harmony. The TDF, COM-B, and BCW mapping suggest that interventions should address motivation and external influences.

## 1. Introduction

High-quality maternal and newborn care is critical for reducing global maternal and newborn mortality and morbidity and supporting positive birth experiences [1]. The World Health Organization’s (WHO) framework for quality of care for maternal and newborn health includes both provision and experience of care domains [2,3]. The experience of care domain emphasises the provision of respectful care throughout the antenatal, childbirth and postnatal periods [2,3]. Health workers play a vital role in ensuring that maternal and newborn care is not only clinically effective but also compassionate, respectful, and responsive to the needs of women, newborns and their families [4].

Health workers providing maternal and newborn care can face significant physical and emotional strain related to their work. There can be high levels of stress, burnout, exhaustion, fatigue, and emotional distress due to the emotionally demanding nature of the work, especially within an overstretched health system [5–11]. This can negatively impact health workers’ well-being, with many health workers reporting that burnout, exhaustion and depression affected their ability to provide high-quality, respectful maternal and newborn care [12,13]. A research modelling pathway between health workers’ mental health and their provision of respectful care also found a significant association between emotional exhaustion, depression and depersonalisation and lower levels of respectful care provision [14]. For example, physically or emotionally exhausted health workers may struggle to show empathy or listen to women’s concerns, or if they feel overwhelmed or stressed, they might rush to end health consultations. The recently released WHO *Compendium on respectful maternal and newborn care* highlights improving and maintaining health workers’ well-being as a critical area for intervention to end mistreatment and achieve respectful maternal and newborn care [15].

Definitions of well-being vary across settings and care contexts [16]. For health workers, the Global Health and Care Workers Compact Framework [17] outlines four main areas to safeguard general health and care workers’ well-being, which are harm prevention, inclusivity, support provision and rights protection. The U.S Surgeon General’s Framework defined workplace mental health and well-being through the principles of harm protection, opportunity for growth, connection and community, mattering at work and work-life harmony [18]. These different conceptualisations of well-being reflect the context-dependent nature of the concept. However, to-date there is no established definition or set of well-being domains specific to health workers working in maternal and newborn care, which creates challenges in developing targeted strategies to support their well-being.

The aim of this qualitative evidence synthesis (QES) was to explore and synthesise key domains of well-being for health workers providing maternal and newborn care, by exploring health workers’ perceptions and experiences. We also aimed to identify potential interventions and implementation strategies to improve health workers’ well-being, guided by theory-driven interventions and implementation strategies.

## 2. Methods

This QES was conducted according to the Cochrane Effective Practice and Organisation of Care template for qualitative evidence synthesis [19] and reported following the Enhancing transparency in reporting the synthesis of qualitative research (ENTREQ) statement [20] (S1 Appendix). The QES protocol has been published (PROSPERO: CRD42024560187).

### 2.1. Type of studies

We included primary studies that used qualitative methodologies for both data collection and analysis, including grounded theory, phenomenology, ethnography, narrative research and participatory action research. Studies that used mixed-methods designs were included where qualitative data could be extracted separately. Non-primary research studies were excluded, including reviews, commentaries, book chapters, systematic or scoping reviews, conference abstracts, and theses. Publications from before 2010 were also excluded, as they may not reflect contemporary maternal and newborn care services. There was no limitation on publication language.

### 2.2. Population of interest

We included health workers who had received formal education and/or training and provided antenatal care, childbirth and postnatal care to women and newborns. This group includes midwives, obstetricians, general doctors, nurses, assistant nurses/midwives, auxiliary midwives, neonatologists and paediatricians [21]. We refer to this group as “health workers providing maternal and newborn care” to reflect the reality that some of these health workers may not be trained only in providing maternal or newborn care, or may not only work in maternity care settings. We recognise that other occupational groups may also be involved to some extent in the delivery of maternal and newborn care.

We excluded studies reporting on (1) health associate professionals who received less or no formal training (e.g., traditional or lay midwives, community health workers), (2) students (e.g., nursing, midwifery and medical students), or (3) sonographers.

### 2.3. Phenomena of interest

We included studies that reported health workers’ perceptions and experiences of well-being while providing routine maternal and newborn care, including general emergency obstetric and newborn care, in any country. This included both positive and negative aspects.

We excluded studies exploring perceptions and experiences of health workers related to (1) their roles outside of clinical care (e.g., academic roles), (2) providing non-routine care or specialist services (e.g., obstetric and neonatal intensive care units, care provision during pandemics, epidemics or humanitarian settings), (3) personal life (e.g., their own pregnancy experiences), and (4) implementing a new program or intervention rather than routine care. We focused on routine maternal and newborn care to capture the daily experiences of health workers in typical care delivery settings as the non-routine care, specialist services, care provision during pandemics/endemics/humanitarian settings involve different skills requirements and conditions that could lead to substantially different findings, and narrowing the scope in this way ensured consistency and focus in this QES.

### 2.4. Search methods for identification of studies

We searched three electronic databases (MEDLINE, CINAHL, MIDIRS) for papers published from 1 January 2010 to the date of search (31 January 2024, search updated on 20 August 2025). The search strategy included terms related to: 1) health workers; 2) maternal and newborn care; 3) well-being; and 4) qualitative research (full search strategy in S2 Appendix). Selection of databases and development of a search strategy were conducted in consultation with a senior information specialist (SM), with a focus on balancing comprehensiveness with efficiency by prioritising highly relevant sources.

### 2.5. Selection of studies

Titles and abstracts from the database search were imported to Covidence (www.covidence.org), and duplicates were removed. Two reviewers (AH, MVC, or NM) independently assessed the eligibility of each reference in Covidence against the inclusion and exclusion criteria. Any disagreement was resolved through discussion between two reviewers or by a third team member to adjudicate any disagreement. Calibration among reviewers was conducted twice, at the beginning of title and abstract screening (first 500 records) and full-text screening (15% of records in the full-text stage) for standardising criteria.

The titles and abstracts of studies published in languages other than those in which the review team is fluent (Bahasa Indonesia, English, French, Portuguese, Spanish, and Turkish) were translated using Google Translate to determine if they were relevant for inclusion. When relevant, the full text was translated using Google Translate (www.translate.google.com) and DeepL (www.deepl.com) and checked by native speakers.

### 2.6. Sampling of studies

We followed best practice in conducting QES to sample our included papers, facilitating deeper engagement with the data and enabling more interpretive analysis. We employed maximum variation purposive sampling to select papers for data extraction [22]. This sampling method helped to limit the number of papers included in the analysis, making the analysis more robust and manageable, while allowing for the broadest possible variation across the included papers to achieve the objectives of the synthesis [22,23].

We adopted an additive approach to sampling, following similar methods to other QES [24]. We used five sampling criteria: data richness, relevance, country-income level, type of health workers, and topics related to well-being. Details of sampling process can be found in S3 Appendix. The sampling process involved two reviewers (AH, NM), and the stages of sampling and decisions were iteratively discussed with the review team. In total, we had 51 sampled papers, reporting 47 discrete studies.

### 2.7. Data extraction

Two reviewers (AH, MVC) extracted data from 51 sampled papers using a form that was designed, developed and pre-tested for this review, including study objectives, study setting, participant characteristics, study designs, data collection and analysis methods, and findings.

### 2.8. Assessing the methodological limitations of included studies

The methodological quality of the included studies was assessed using CochrAne qualitative Methodological LimitatiOns Tool (CAMELOT) [25]. CAMELOT incorporates principles of qualitative research to assess methodological strengths and limitations, focusing and reflecting on the appropriateness of fit between domains [25]. Two reviewers (AH, MVC) critically assessed the sampled papers independently against the CAMELOT criteria during data extraction. Any disagreements were resolved through discussion between reviewers. The assessment results were not used as a basis for exclusion but formed part of the confidence assessment for each review finding. Detailed assessments of methodological limitations of the sampled studies are available in S4 Appendix.

### 2.9. Data management, analysis and synthesis

A thematic synthesis approach was used to analyse and synthesise extracted data [26]. First, the extracted data were coded line-by-line, then organised first into descriptive themes, and then into analytical themes to generate interpretive explanations of the findings [26]. Themes were reviewed and refined through discussions with other review team members.

We adopted a mixed inductive-deductive analysis approach to develop the domains using existing frameworks of health workers’ well-being. As there is no framework specifically for health workers providing maternal and newborn care, we used two frameworks of health workers’ well-being: Global health and care workers compact framework [17] and the U.S. Surgeon General’s Framework for Workplace Mental Health and Well-Being [18]. We integrated these frameworks, then coded the final synthesis findings into this integrated framework. We created new domains if the findings did not align with the integrated framework. The review team reviewed and refined the domains through discussions. Qualitative analysis was conducted using NVivo [27].

### 2.10. Assessing confidence in synthesis findings

We assessed confidence in the synthesis findings by using the GRADE-CERQual (Confidence in the Evidence from Reviews of Qualitative research) approach [28]. GRADE-CERQual assessments were conducted based on four components:

1) Methodological limitations of included studies: the extent to which there are problems in the design or conduct of the primary studies supporting a review [29]. We used the results of the CAMELOT assessment to inform confidence in this component in each of the synthesis findings.
2) Coherence of the finding: an assessment of how clear and cogent the fit is between the data from the primary studies and the review finding [30].
3) Adequacy of data contributing to a review finding: the degree of richness and quantity of data supporting a review finding [31].
4) Relevance of the included studies to review question: the extent to which the body of evidence from the primary studies supporting a review finding is applicable to the context specified in the review question [32].

Two reviewers (AH, MVC) assessed each component by the level of concerns (no or very minor, minor, moderate, and serious) [28–33]. Then, we made a judgment about overall confidence in each finding via consensus, considering level of concern in each component. The overall confidence was categorised as high, moderate, low or very low [33]. All findings started as high confidence and were downgraded if there were important concerns regarding any of the GRADE-CERQual components.

### 2.11. Mapping qualitative synthesis to behaviour change frameworks

We mapped our synthesis findings to the Theoretical Domains Framework (TDF) of behaviour change [34] and the Capability, Opportunity and Motivation Behavior (COM-B) framework [35] to identify and provide a theory-informed basis for the development of potential implementation strategies to improve health workers’ well-being. Once findings were mapped, we developed potential implementation strategies based on the Behaviour Change Wheel [35], to tailor strategies based on the QES findings and how they might influence intervention functions and policy categories. The initial mapping was conducted by AH, validated by RIZ, and finalised through discussion with MB.

### 2.12. Consultations. with health workers

To ensure that the synthesis findings, domains of well-being and potential implementation strategies were relevant and clear to health workers, we conducted individual and group sense-checking consultations with health workers who provide routine maternal and newborn care globally. We recruited these health workers through our existing professional networks. We conducted four individual and one group sense-checking consultations with 18 health workers (3 obstetricians, 1 neonatologist, 14 midwives) in the African and Asia-Pacific regions (Australia, Bhutan, Ethiopia, Fiji, India, Indonesia, Kenya, New Zealand, Pakistan, Samoa, and Solomon Islands). Each consultation was conducted within 60 minutes and audio-recorded. During the consultation, we asked health workers to review a draft of the findings, domains, and strategies, and to indicate whether the findings accurately reflect the context of health workers in their setting. We also sought their suggestions for improving health workers’ well-being. All consultations were conducted online, and two reviewers attended the consultations (AH, MVC, or RIZ).

### 2.13. Review author reflexivity

The author team represents diverse personal, geographical, social, and professional backgrounds, which might have influenced the conduct and interpretation of this QES. The team consisted of seven authors based in Melbourne, Australia; two in Geneva, Switzerland; one in Antwerp, Belgium; two in the United States; one in the United Kingdom, and one who split their time between Melbourne and Lombok, Indonesia. The team has worked on various projects or research aimed at improving maternal and newborn health across diverse contexts, such as Brazil, Ethiopia, Ghana, Guinea, Kenya, India, Indonesia, Myanmar, Malawi, Nigeria, and Papua New Guinea. Our disciplinary expertise encompasses public health, social sciences, epidemiology, midwifery, medicine and evidence synthesis, with extensive experience in applying qualitative methodologies to maternal and newborn health research. The team also included a PhD student trained in qualitative research and two early-career researchers. Several authors had direct professional experience as health workers providing maternal and newborn care, while others had collaborated closely with health workers in previous research. This background provided us with a nuanced understanding of the context, practices, and challenges faced by health workers in this field.

## 3. Results

**Fig 1:**
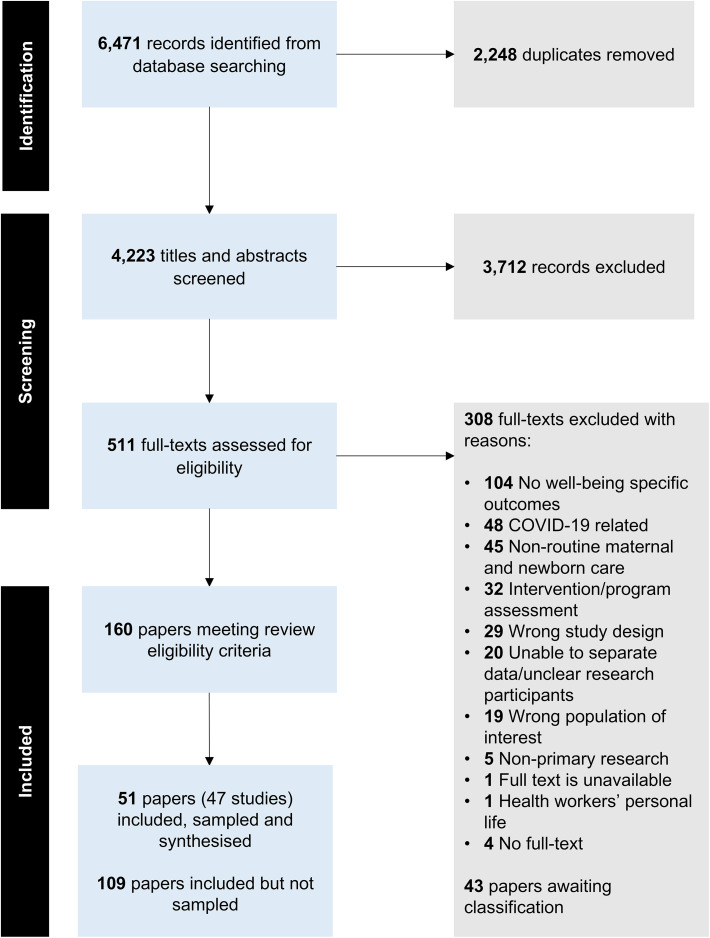
PRISMA flow diagram.

We identified 6,471 records from database searches. Of the 160 papers that met the inclusion criteria from the original search, we sampled 51 papers from 47 studies for inclusion in the analysis (see S5 Appendix. Characteristics of the sampled studies). The search update in August 2025 identified an additional 43 papers. We chose not to sample these papers or extract characteristics as the existing sample of 51 papers already provided sufficient depth and rich data and covered key concepts of health workers’ well-being (see S6 Appendix for papers awaiting classification). Fig 1 presents the study selection process in a PRISMA flow diagram.

### 3.1. Description of sampled papers

Forty papers used qualitative designs [36–75] and 11 papers used mixed-methods study designs [76–86]. All papers were published in English, except one published in French [38].

The 51 papers present research conducted in 23 countries and five geographic regions. Eighteen papers were from Europe: Belgium [80], Ireland [50,51,64], Norway [39], Sweden [72,73], Switzerland [83], the Netherlands [71,80], Turkey [46,70] and the United Kingdom [52,57,62,68,81,85]. Fourteen papers were from Africa: Benin [59], Burkina Faso [59], Ghana [36,37,48,49,58,61], Malawi [43], Namibia [66], South Africa [60] and Tanzania [41,42,44,65]. Eight papers were from The Americas and Western Pacific each: Australia [53,63,67,84], Canada [47,79], China [54], New Zealand [45,75,82] and the United States [40,74,76–78,86]. Three papers were from the Eastern Mediterranean: Iran [55,69], Israel [56] and Morocco [38]. Most studies were conducted in high-income countries (31/51 papers) [39,40,45,47,50–53,56,57,62–64,67,68,71–86], with 20 conducted in low- and middle-income countries (LMICs) [36–38,41–44,46,48,49,54,55,58–61,65,66,69,70].

Most papers reported the perspectives of health workers working in health facility settings [36–39,41–44,46–56,58,60–69,72–74,80–83,86], and included perspectives of midwives (44, 71% [36–39, 41–63, 66–73,75,76,79–84,86]), nurses (9, 15% [39,40,43,61,65,74,77,78,83]), obstetricians (6, 10% [39,61,64,72,73,85]) and general practitioners (1, 2% [83]). Table 1 reports the summary characteristics of the sampled papers.

**Table 1:** Characteristics of sampled papers (n = 51).

Characteristics	n	%
**Year of publication**		
2020-2024	24	47%
2015-2019	19	37%
2010-2014	8	16%
**Research design**		
Qualitative only	40	78%
Mixed-methods	11	22%
**Study region**		
Europe	18	35%
Africa	14	27%
The Americas	8	16%
Western Pacific	8	16%
Eastern Mediterranean	3	6%
**Country income level**		
High-income	31	61%
Middle-income	19	37%
Low-income	1	2%
**Study setting** ^a^		
Facility-based	40	63%
Community-based	10	16%
Not specified	13	21%
**Type of health workers** ^b^		
Midwives	44	71%
Nurses	9	14%
Obstetricians	6	10%
Nurse-midwives	2	3%
General practitioners	1	2%

^a^Eight papers conducted study in multiple types of health facilities [47,52,62,67,80–82,86]

^b^Seven papers reported perspectives from multiple health workers [39,40,43,61,72,73,83].

### 3.2. Qualitative synthesis

**Fig 2:**
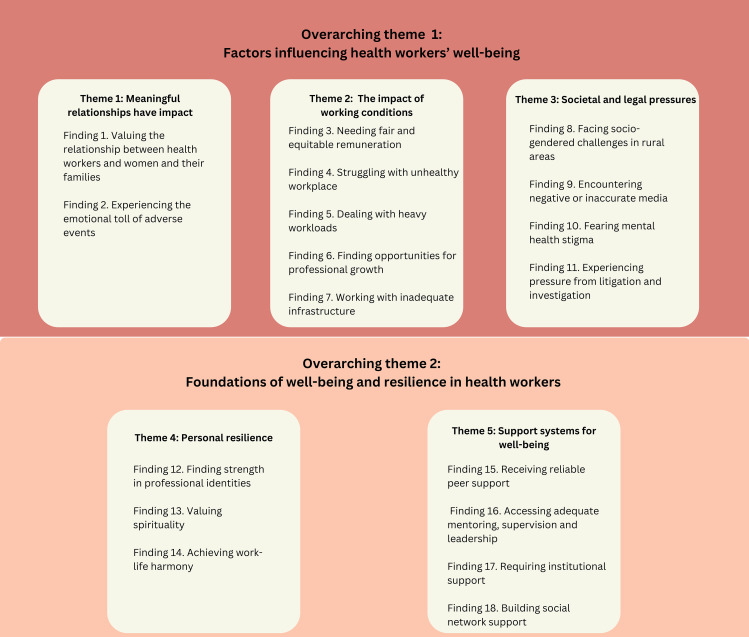
Themes derived from the qualitative synthesis.

Table 2 presents the summary of qualitative findings and GRADE-CERQual assessments (see full evidence profile in S7 Appendix). Eighteen synthesis findings were developed through the qualitative synthesis and were categorised into five main themes: 1) duality of caregiving, 2) working conditions, 3) societal and legal pressures, 4) personal resilience, and 5) support systems for well-being. These five themes were further grouped into two overarching themes: 1) factors influencing health workers’ well-being (Finding 1–11) and 2) foundations of well-being and resilience in health workers (Finding 12–18) (Fig 2).

**Table 2:** Summary of qualitative findings.

Findings	Summary of qualitative findings	Contributing papers	Overall CERQual assessment	Explanation of overall assessment
**Overarching Theme 1: Factors influencing health workers’ well-being**				
**Theme 1: Meaningful relationships have impact**				
1	**Valuing the relationship between health workers and women and their families.** Health workers valued the trust and relationships they built with women and their families. These relationships led to both positive emotional experiences and, at times, significant emotional strain.	(34, 36, 38, 42, 43, 45, 49-53, 56-58, 60, 62, 64, 67, 69, 70, 75-77, 80, 81, 83)	Moderate confidence	No or very minor concerns on coherence, minor concerns on relevance (13 papers with indirectly relevant aim), minor concerns on adequacy (26 of 51 papers contributed; 15 moderately thick and 11 thin data), moderate concerns on methodological limitations (fit between research design and researchers, fit between research conduct and researchers, fit between research design and research conduct, fit between research conduct and research aim/questions, fit between research conduct and context, fit between research design domains and research aim/ questions)
2	**Experiencing the emotional toll of adverse events.** Maternal and neonatal complications or deaths were traumatic events for health workers, with significant negative and long-lasting impacts on their mental and physical health. Health workers often questioned their care decisions, felt a heavy sense of responsibility for adverse outcomes, experienced stressful flashbacks, and had difficulty coping with their emotions.	(34, 36-38, 40, 43, 44, 46, 47, 49, 51, 53-58, 62, 64-66, 70, 72, 75-78, 81-83)	Moderate confidence	No or very minor concerns on coherence, no or very minor concerns on adequacy, minor concerns on relevance (12 papers with indirectly relevant aim), moderate concerns on methodological limitations (fit between research design domains and researchers, fit between research conduct domains and researchers, fit between research design domains and context, fit between research conduct domains and context, fit between research design domains and research conduct domain, fit between research conduct domains and research aim/questions, fit between research conduct domains and stakeholders)
**Theme 2: The impact of working conditions**				
3	**Needing fair and equitable remuneration.** Fair and equitable remuneration across different professions and service areas (e.g., rural or urban) was crucial for midwives and nurses to feel valued and respected. When pay was unfair and inequitable, they felt unrecognised, undervalued, frustrated and demotivated.	(35, 42, 57, 63, 77)	Low confidence	No or very minor concerns on coherence, minor concerns on methodological limitations (fit between research design domains and researchers, fit between research conduct domains and researchers, fit between research design domains and research conduct domain), moderate concerns on relevance (4 papers with indirectly relevant aim, all papers only represented two regions), serious concerns on adequacy (5 of 51 papers contributed; 3 moderately thick and 2 thin data)
4	**Struggling with unhealthy workplace culture.** Some health workers perceived that an unhealthy workplace culture contributed to their experiences of mental distress, powerlessness, isolation, burnout and poor job performance and satisfaction.	(37, 38, 40, 41, 43-45, 48, 50, 51, 54-68, 70-74, 77, 81, 83, 84)	Moderate confidence	No or very minor concerns on coherence, no or very minor concerns on adequacy, minor concerns on relevance (17 papers with indirectly relevant aim), moderate concerns on methodological limitations (fit between research design domains and researchers, fit between research design domains and context, fit between research conduct domains and researchers, fit between research conduct domains and context, fit between research design domains and stakeholders, fit between research conduct domains and stakeholders, fit between research design domains and research conduct domain, fit between research conduct domains and research aim/questions)
5	**Dealing with heavy workloads.** Heavy workloads, exacerbated by staff shortages, inadequate skills, and poor workload management, resulted in emotional distress, burnout, and physical injuries. These negative emotional experiences were further intensified by the inability to take leave or by feelings of guilt associated with taking time off, as it could place additional burdens on their colleagues.	(35, 41, 42, 44, 45, 48, 49, 51, 52, 55, 56, 58, 61, 63, 64, 71, 73, 74, 77, 81, 82)	Moderate confidence	No or very minor concerns on coherence, no or very minor concerns on adequacy, minor concerns on relevance (10 papers with indirectly relevant aim), moderate concerns on methodological limitations (fit between research design domains and researchers, fit between research conduct domains and researchers, fit between research design domains and research conduct domain, fit between research conduct domains and stakeholders, fit between research conduct domains and research aim/questions)
6	**Finding opportunities for professional growth.** Opportunities for growth, including training, further education, and career advancement, were positive motivators for midwives. However, their access to these opportunities was limited when health facilities faced staff shortages or implemented unpredictable scheduling systems.	(35, 42, 52)	Very low confidence	No or very minor concerns on coherence, minor concerns on methodological limitations (fit between research design domains and researchers, fit between research conduct domains and researchers, fit between research design domains and research conduct domain), serious concerns on relevance (all papers with indirectly relevant aim, perspectives came only from midwives in LMICs), serious concerns on adequacy (3 of 51 papers contributed; 2 moderately thick and 1 thin data)
7	**Working with inadequate infrastructure.** Insufficient infrastructure in health facilities, including the absence of clean running water and shortages of essential equipment and supplies, heightened the risk of infections and physical injuries among midwives and nurses, leading to frustration as it hindered their ability to deliver high-quality care to women and their families. Inadequate infrastructure also negatively impacted midwives and nurses’ physical and psychological health.	(35, 40, 42, 56-58, 61, 63, 64, 74, 79, 82).	Moderate confidence	No or very minor concerns on coherence, minor concerns on relevance (7 papers with indirectly relevan aim, represented midwives perspectives only), moderate concerns on adequacy (12 of 51 papers contributed; 6 moderate and 6 thin data), moderate concerns on methodological limitations (fit between research design domains and research conduct domain, fit between research conduct domains and research aim/questions, fit between research design domains and researchers, fit between research conduct domains and researchers).
**Theme 3: Societal and legal pressures**				
8	**Facing socio-gendered challenges in rural areas.** Rural postings provided midwives and nurses with opportunities for quicker career advancement, but they also presented challenges, including poor living conditions, inadequate housing, limited access to quality education, and safety risks. For female health workers, these risks were even more pronounced due to sexist and gendered power dynamics, which could adversely impact their career progression and transfer requests.	(35, 56, 57, 63, 80)	Low confidence	No or very minor concerns on coherence, moderate concerns on relevance (all papers with indirectly relevant aim, represented two regions), moderate concerns on methodological limitations (fit between research design domains and research conduct domain, fit between research design domains and research aim/ questions, fit between research conduct domains and research aim/ questions, fit between research design domains and researchers, fit between research conduct domains and researchers), serious concerns on adequacy (5 of 51 papers contributed; 4 moderately thick and 1 thin data)
9	**Encountering negative or inaccurate media portrayals.** Media coverage blaming midwives and obstetricians for maternal or newborn injury or death made health workers feel upset, frustrated and powerless. Health workers felt these reports were often inaccurate, but they were unable to share their perspectives due to professional confidentiality and limited support from their employers.	(62, 64, 70, 71)	Low confidence	No or very minor concerns on coherence, minor concerns on methodological limitations (fit between research design domains and researchers, fit between research design domains and research conduct domain, fit between research conduct domains and research aim/questions, fit between research conduct domains and researchers), moderate concerns on relevance (3 papers with indirectly relevant aim, represented two regions), serious concerns on adequacy (4 of 51 papers contributed; 2 thick and 2 thin data)
10	**Fearing mental health stigma.** Mental health stigma related to both sharing experiences and accessing support meant some health workers were reluctant to seek help for themselves. Concerns about a lack of confidentiality when seeking mental health care further contributed to this hesitation, leading to many health workers choosing to consult with senior colleagues rather than pursue professional support.	(59, 69)	Low confidence	No or very minor concerns on coherence, moderate concerns on relevance (all papers represented 2 countries), moderate concerns on methodological limitations (fit between research design domains and research conduct domain, fit between research conduct domains and context, fit between research conduct domains and research aim/questions), serious concerns on adequacy (2 of 51 papers contributed with moderately thick data)
11	**Experiencing pressure from litigation and investigation.** Clinical audits, lawsuits, and professional investigations affected health workers’ mental and physical health, with the impact worsened by perceived unfair processes. During these proceedings, health workers reported that they did not receive adequate legal and emotional support from their employers.	(34, 36, 38, 44, 56, 64, 66, 70, 71, 83, 84)	Moderate confidence	No or very minor concerns on coherence, minor concerns on relevance (4 papers with indirectly relevant aim), moderate concerns on adequacy (11 of 51 papers contributed; 6 moderately thick and 5 thin data), moderate concerns on methodological limitations (fit between research design domains and researchers, fit between research conduct domains and researchers, fit between research design domains and context, fit between research conduct domains and context, fit between research design domains and research conduct domain, fit between research conduct domains and research aim/questions, fit between research design domains and research aim/questions)
**Overarching Theme 2: Foundations of well-being and resilience in health workers**				
**Theme 4: Personal resilience**				
12	**Finding strength in professional identities.** Professional identities were crucial in promoting resilience. Professional identities were shaped by cognitive, emotional, and relational dimensions, involving autonomy, belonging, fulfilment, achievement, responsibility, passion, compassion for patients, confidence in their abilities, and self-efficacy.	(40, 43, 45, 49-57, 60, 62, 64-69, 72, 73, 77, 80-82)	Moderate confidence	No or very minor concerns on coherence, no or very minor concerns on adequacy, minor concerns on relevance (13 out of 26 papers with indirectly relevant aim), moderate concerns on methodological limitations (fit between research design domains and stakeholders, fit between research design domains and researchers, fit between research design domains and research conduct domain, fit between research conduct domains and stakeholders, fit between research conduct domains and researchers, fit between research conduct domains and context, fit between research design domains and research aim/questions, fit between research conduct domains and research aim/questions, fit between research design domains and context)
13	**Valuing spirituality.** Spiritual beliefs and practices enabled some health workers to cope with significant challenges, such as maternal and neonatal deaths, which fostered resilience and provided a sense of purpose.	(34, 36, 39, 58, 67, 72, 75, 76)	Moderate confidence	No or very minor concerns on coherence, minor concerns on relevance (3 papers with indirectly relevant aim), moderate concerns on adequacy (8 of 51 papers contributed; 5 moderate and 3 thin data), moderate concerns on methodological limitations (fit between research design domains and researchers, fit between research conduct domains and researchers, fit between research design domains and research conduct domain, fit between research conduct domains and research aim/questions, fit between research design domains and context, fit between research conduct domains and context),
14	**Achieving work-life harmony.** Achieving work-life harmony was seen as a potential solution to reduce burnout and alleviate mental and physical distress among health workers, ultimately helping to build their resilience. However, several factors hindered this, including unclear boundaries between personal and professional lives, expectations to be constantly available, physical injuries, personal caregiving responsibilities, inadequate workload management, exposure to adverse events, and long commutes to work.	(36, 38, 41, 42, 44, 46, 47, 49, 51-57, 61, 72-74, 77-82, 84)	Moderate confidence	No or very minor concerns on coherence, no or very minor concerns on relevance, no or very minor concerns on adequacy, moderate concerns on methodological limitations (fit between research design domains and researchers, fit between research design domains and research conduct domain, fit between research conduct domains and researchers, fit between research design domains and stakeholders, fit between research conduct domains and stakeholders, fit between research conduct domains and context, fit between research design domains and research aim/questions, fit between research conduct domains and research aim/questions, fit between research design domains and context)
**Theme 5: Support systems for well-being**				
15	**Receiving reliable peer support.** Support from colleagues and within the professional community was a crucial coping strategy for addressing workplace stress, exhaustion, and burnout among health workers. When health workers had colleagues with whom they could share their feelings and experiences, seek advice, receive reassurance, or even a comforting hug, it helped alleviate feelings of isolation and ensured a sense of safety in their practice.	(36, 37, 43-45, 48, 51-55, 61, 62, 66, 67, 69-75, 78, 83)	Moderate confidence	No or very minor concerns on coherence, no or very minor concerns on adequacy, minor concerns on relevance (24 papers represented 11 high income countries), moderate concerns on methodological limitations (fit between research design domains and researchers, fit between research design domains and research conduct domain, fit between research conduct domains and researchers, fit between research design domains and stakeholders, fit between research conduct domains and stakeholders, fit between research design domains and context, fit between research conduct domains and context, fit between research conduct domains and research aim/questions)
16	**Accessing adequate mentoring, supervision and leadership.** Health workers described that adequate mentoring and supervision, along with strong leadership, were crucial for creating a healthier work environment, including managing difficult workplace situations and reducing workplace stress.	(35-37, 40, 41, 43, 44, 48-51, 53-56, 58, 59, 61, 63, 66, 70-74, 76, 77, 83, 84)	Moderate confidence	No or very minor concerns on coherence, minor concerns on relevance (11 papers with indirectly relevant aim), minor concerns on adequacy (29 of 51 papers contributed; 21 thick and 8 thin data), moderate concerns on methodological limitations (fit between research design domains and researchers. fit between research conduct domains and researchers, fit between research design domains and research conduct domain, fit between research conduct domains and research aim/questions, fit between research design domains and stakeholders, fit between research conduct domains and stakeholders, fit between research conduct domains and context, fit between research design domains and research aim/questions
17	**Requiring institutional support.** Health workers required institutional support from their workplace, including mental health, emotional support and guidance. Many workplaces failed to provide this support, and when available, the support often focused on investigating the clinical aspects of the event or criticising health workers, rather than addressing the emotional needs of health workers.	(34, 36, 37, 39, 43-45, 48, 51, 53, 54, 56, 58, 59, 62, 65, 66, 71-73, 77, 82, 83)	Moderate confidence	No or very minor concerns on coherence, no or very minor concerns on relevance, minor concerns on adequacy (24 of 51 papers contributed; 16 moderately thick and 8 thin data), moderate concerns on methodological limitations (fit between research design domains and researchers, fit between research conduct domains and researchers, fit between research design domains and research conduct domain, fit between research conduct domains and research aim/questions, fit between research design domains and stakeholders, fit between research conduct domains and stakeholders, fit between research design domains and research aim/questions, fit between research conduct domains and context)
18	**Building social network support.** Family and friends played a dual role in supporting health workers. Some health workers perceived that family and friends served as valuable support systems when facing workplace challenges, while others avoided or found limited support in their family and friends.	(36, 37, 43, 44, 49, 52-55, 58, 62, 66, 67, 72, 77, 78)	Moderate confidence	No or very minor concerns on coherence, minor concerns on relevance (majority of papers represented countries in EURO region), moderate concerns on adequacy (16 of 51 papers contributed; 6 moderate and 10 thin data), moderate concerns on methodological limitations (fit between research design domains and researchers, fit between research design domains and research conduct domain, fit between research conduct domains and research aim/questions, fit between research conduct domains and researchers, fit between research design domains and stakeholders, fit between research conduct domains and stakeholders, fit between research conduct domains and context, fit between research design domains and context)

#### 3.2.1. Factors influencing health workers’ well-being.

**3.2.1.1. Meaningful relationships have impact.** Thirty-eight papers reported the influence that the relationships between health workers and women and their families had on the health workers, and how these relationships impacted them, both positively and negatively [36,38–40,42,44–49,51–60,62,64,66–69,71,72,74,77–80,82–85].

*Finding 1. Valuing the relationship between health workers and women and their families* **Health workers valued the trust and relationships they built with women and their families. These relationships led to both positive emotional experiences and, at times, significant emotional strain** (Moderate confidence) [36,38,40,44,45,47,51–55,58–60,62,64,66,69,71,72,77–79,82,83,85].

Supporting women’s choices during childbirth or breastfeeding and witnessing women’s joy after birth were powerful sources of fulfilment and motivation for health workers [38,52,69,77,83]. However, following poor outcomes, health workers often experienced grief and sadness. Maintaining communication with women and families after such events helped mitigate this emotional burden [51,60,64,66,71,78]. Stress increased when women disregarded health workers’ advice [40,54,66,71,72,79], when they faced hostility, were blamed for negative outcomes, or experienced verbal and physical abuse from women and their families [44,58,59,72].

*Finding 2. Experiencing the emotional toll of adverse events* **Maternal and neonatal complications or deaths were traumatic events for health workers, with significant negative and long-lasting impacts on their mental and physical health. Health workers often questioned their care decisions, felt a heavy sense of responsibility for adverse outcomes, experienced stressful flashbacks, and had difficulty coping with their emotions** (Moderate confidence) [36,38–40,42,45,46,48,49,51,53,55–60,64,66–68,72,74,77–80,83–85].

For some, traumatic events could lead to positive personal and professional reflection [45,46,52,55,80], building emotional resilience [38,39,41,72,77] and appreciation for life [77]. However, others resorted to harmful coping mechanisms, such as increased alcohol consumption or social withdrawal [36,38–40,49,51,53,55–57,64,72,78,80].

**3.2.1.2 The impact of working conditions.** Forty papers showed that health workers perceived their working conditions as significantly impacting their physical and mental well-being [37,39,40,42–47,50–54,56–70,72–76,79,81,83–86]. Some health workers faced numerous challenges, including unfair remuneration, unhealthy workplace culture, heavy workloads, limited growth opportunities, and inadequate infrastructure, leading to distress, burnout, powerlessness, and reduced job performance and satisfaction.

*Finding 3. Needing fair and equitable remuneration* **Fair and equitable remuneration across different professions and service areas (e.g., rural or urban) was crucial for midwives and nurses to feel valued and respected. When pay was unfair and inequitable, they felt unrecognised, undervalued, frustrated and demotivated** (Low confidence) [37,44,59,65,79].

Unfair and inequitable remuneration was mainly reported in papers from Africa, where some midwives and nurses working in rural areas felt inadequately compensated by their employers, while doctors or more senior health workers working in the same region received rural incentive allowances [37,59,65]. This situation was exacerbated by a lack of transparency and fairness in the compensation system [65].

*Finding 4. Struggling with unhealthy workplace culture* **Some health workers perceived that an unhealthy workplace culture contributed to their experiences of mental distress, powerlessness, isolation, burnout and poor job performance and satisfaction** (Moderate confidence) [39,40,42,43,45–47,50,52,53,56–70,72–76,79,83,85,86].

An unhealthy workplace culture was characterised by hierarchical dynamics, unfair treatment, discrimination, and inadequate support [40,45,50,53,56,57,59,64,69,70,78,79,83]. Inadequate support manifested in policies neglecting health workers’ physical and emotional health, judgmental attitudes, unreliable colleagues, and insufficient channels to voice concerns [39,45,46,50,52,55–57,59,64–66,69,73,74,86]. This culture normalised injuries, trauma, and burnout, [45,50,53,60,61,63,72,75,76,79]. Health workers struggled to recognise and address their own emotional and physical health, often suppressing their emotions and avoiding breaks for fear of appearing weak or vulnerable [45,53,63,68]. When they did sustain physical injuries, the workplace culture discouraged them from filing compensation claims, or there was no system in place to compensate for their injuries [58,63].

*Finding 5. Dealing with heavy workloads* **Heavy workloads, exacerbated by staff shortages, inadequate skills, and poor workload management, resulted in emotional distress, burnout, and physical injuries. These negative emotional experiences were further intensified by the inability to take leave or by feelings of guilt associated with taking time off, as it could place additional burdens on their colleagues** (Moderate confidence) [37,43,44,46,47,50,51,53,54,57,58,60,63,65,66,73,75,76,79,83,84].

Health workers viewed their roles as already emotionally and physically heavy and staff shortages contributed to greater workloads and limited breaks [37,43,53,57,65,66]. Having colleagues with inadequate skills further heightened this burden, especially when care was complex [53]. On-call systems, which aimed to manage health workers’ workload, led to increased anxiety among some due to their inherent uncertainty [54,79]. Moreover, a lack of awareness about burnout symptoms led health workers to confuse these symptoms with other illnesses, preventing them from seeking the necessary support, ultimately exacerbating their burnout [51,75].

*Finding 6. Finding opportunities for professional growth* **Opportunities for growth, including training, further education, and career advancement, were positive motivators for midwives. However, their access to these opportunities was limited when health facilities faced staff shortages or implemented unpredictable scheduling systems** (Very low confidence) [37,44,54].

Health workers often had little time available to participate in activities for their professional development [37,44]. In particular, midwives working in rural areas had less access to in-service training opportunities compared to their urban counterparts [37].

*Finding 7. Working with inadequate infrastructure* **Insufficient infrastructure in health facilities, including the absence of clean running water and shortages of essential equipment and supplies, heightened the risk of infections and physical injuries among midwives and nurses, leading to frustration as it hindered their ability to deliver high-quality care to women and their families. Inadequate infrastructure also negatively impacted midwives and nurses’ physical and psychological health** (Moderate confidence) [37,42,44,58–60,63,65,66,76,81,84].

Inadequate infrastructure compelled midwives and nurses to adopt long hours in awkward or unergonomic positions, increasing their risk of physical injuries [37,59,63]. For instance, due to a shortage of hospital beds, women had to lie on mattresses on the floor, and midwives had to bend and squat while providing care [37]. For staff working long shifts often forced health workers not to rest or seek out vacant patient rooms [60,76].

**3.2.1.3. Societal and legal pressures.** Eighteen papers reported social, gender, and legal factors influencing health workers’ physical and mental health [36–38,40,46,58,59,61,64–66,68,71–73,82,85,86]. Findings 8–11 highlight various social issues, including poor and unsafe living conditions in rural areas, gendered power dynamics in rural health facilities hindering the career progression of female health workers, negative media coverage, and mental health stigma. Legal pressures such as clinical audits, lawsuits, and professional investigations also affected health workers’ physical and mental health.

*Finding 8. Facing socio-gendered challenges in rural areas* **Rural postings provided midwives and nurses with opportunities for quicker career advancement, but they also presented challenges, including poor living conditions, inadequate housing, limited access to quality education, and safety risks. For female health workers, these risks were even more pronounced due to sexist and gendered power dynamics, which could adversely impact their career progression and transfer requests** (Low confidence) [37,58,59,65,82].

Female health workers in rural areas often experienced sexual harassment from influential male figures such as high-ranking officials or local leaders, risking professional penalties, including job loss or ostracism, if they rejected the harassment [59]. Requests for transfer to safer areas were often determined by marital status (e.g., married women were less likely to be transferred) rather than professional competence, further restricting professional opportunities and exacerbating risk of further harassment [59].

*Finding 9. Encountering negative or inaccurate media portrayals* **Media coverage blaming midwives and obstetricians for maternal or newborn injury or death made health workers feel upset, frustrated and powerless. Health workers felt these reports were often inaccurate, but they were unable to share their perspectives due to professional confidentiality and limited support from their employers** (Low confidence) [64,66,72,73].

Midwives and obstetricians felt that there was a significant mismatch between public perceptions and the reality of their work, resulting in blame when injuries or deaths occurred [64]. They perceived that the public held an unrealistic belief that pregnancy and childbirth were entirely risk-free [64]. Media coverage further heightened concerns about their professional reputation and family safety [64,66].

*Finding 10. Fearing mental health stigma* **Mental health stigma related to both sharing experiences and accessing support meant some health workers were reluctant to seek help for themselves. Concerns about a lack of confidentiality when seeking mental health care further contributed to this hesitation, leading to many health workers choosing to consult with senior colleagues rather than pursue professional support** (Low confidence) [61,71].

For example, in Ghana, health workers felt they could not seek mental health support for themselves due to stigma [61]. This led to fear of admitting that they had mental health issues [61].

*Finding 11. Experiencing pressure from litigation and investigation* **Clinical audits, lawsuits, and professional investigations affected health workers’ mental and physical health, with the impact worsened by perceived unfair processes. During these proceedings, health workers reported that they did not receive adequate legal and emotional support from their employers** (Moderate confidence) [36,38,40,46,58,66,68,72,73,85,86].

Many expected their employers to provide active legal representation to defend them actively or offer emotional support, but some reported the legal and emotional support they received was inadequate or fell short of their expectations [46,68]. Due to clinical audits, lawsuits or professional investigations, some experienced stress, guilt, anxiety, fear of blame, insomnia, heart issues, and stomach ulcers [36,38,40,57,58,66,68,85,86].

#### 3.2.2. Foundations of well-being and resilience in health workers.

**3.2.2.1. Personal resilience.** Forty-three papers reported on the factors that contribute to resilience among health workers in challenging work environments [36,38,40–49,51–60,62–64,66–71,74–84,86]. Maintaining professional identities, practising spirituality, and achieving work-life balance were fundamental to the personal resilience of health workers (Findings 12 – 14).

*Finding 12. Finding strength in professional identities* **Professional identities were crucial in promoting resilience. Professional identities were shaped by cognitive, emotional, and relational dimensions, involving autonomy, belonging, fulfilment, achievement, responsibility, passion, compassion for patients, confidence in their abilities, and self-efficacy** (Moderate confidence) [42,45,47,51–59,62,64,66–71,74,75,79,82–84].

Autonomy in decision-making allowed health workers to feel confident and in control during practice [57,69,83]. A sense of belonging to their professional groups provided emotional support and reinforced their connection to their work [57]. A sense of responsibility for caring, passion towards the work and compassion for patients influenced their working enthusiasm and motivation [42,51,54,58,59,69,75], while self-efficacy reinforced their belief in making a positive difference [57]. However, limited decision-making power, hierarchical communication, or conflicts between professional values and facility policies could undermine professional identities, leading to stress and a diminished sense of professional self [45,47,52,53,57,62,67,68,74,79,83].

*Finding 13. Valuing spirituality***. Spiritual beliefs and practices enabled some health workers to cope with significant challenges, such as maternal and neonatal deaths, which fostered resilience and provided a sense of purpose** (Moderate confidence) [36,38,41,60,69,74,77,78].

These beliefs and practices served as immediate emotional buffers, helping overcome stress, shock, or sadness [36,41,60,78]. They were a safeguard against feelings of helplessness or overwhelming responsibility, stemming from the belief in a benevolent higher power [36,41,77]. Spiritual beliefs also offered a solution, as the notion of a higher power that can “orchestrate measures for the good” implies that faith can help resolve or improve difficult situations [36,69,74,77]. Spiritual practices included prayers, fasting, requesting prayers from religious leaders, and reading, recitation or recourse [36,41,60,69].

*Finding 14. Achieving work-life harmony* **Achieving work-life harmony was seen as a potential solution to reduce burnout and alleviate mental and physical distress among health workers, ultimately helping to build their resilience. However, several factors hindered this, including unclear boundaries between personal and professional lives, expectations to be constantly available, physical injuries, personal caregiving responsibilities, inadequate workload management, exposure to adverse events, and long commutes to work** (Moderate confidence) [38,40,43,44,46,48,49,51,53–59,63,74–76,79–84,86].

The expectations to be constantly available and exposure to adverse events made it difficult for many health workers to relax after their work, as they ruminated about their patients or what they could have done differently, which impacted their sleep and family relationships [40,43,44,51,53,55,63,75,76,82,83]. Physical injuries limited the personal time of health workers and their time for caring for their children [63], leading to increased stress levels at work. For some health workers, achieving work-life harmony could become a source of stress if not managed effectively [86].

**3.2.2.2 Support systems for well-being.** Forty papers reported that health workers believed that support systems were beneficial for them [36–39,41–43,45–47,50–58,60,61,63–65,67–69,71–80,84–86]. Support systems included their colleagues, as well as adequate mentoring and leadership and adequate institutional support when they faced challenges at work. Health workers also valued support from their family and friends (Findings 15–18).

*Finding 15. Receiving reliable peer support. S***upport from colleagues and within the professional community was a crucial coping strategy for addressing workplace stress, exhaustion, and burnout among health workers. When health workers had colleagues with whom they could share their feelings and experiences, seek advice, receive reassurance, or even a comforting hug, it helped alleviate feelings of isolation and ensured a sense of safety in their practice** (Moderate confidence) [38,39,45–47,50,53–57,63,64,68,69,71–77,80,85].

While some health workers preferred to confide in colleagues from the same clinical background [53,68,75,80]. Others valued the benefits of interprofessional advice [69].

*Finding 16. Accessing adequate mentoring, supervision and leadership* **Health workers described that adequate mentoring and supervision, along with strong leadership, were crucial for creating a healthier work environment, including managing difficult workplace situations and reducing workplace stress** (Moderate confidence) [37–39,42,43,45,46,50–53,55–58,60,61,63,65,68,72–76,78,79,85,86].

Key components were offering guidance on job skills [37,42,65], providing emotional and psychological support [45,46,50,56–58,63,68,72,73,85], recognising and appreciating employees’ efforts [38,44,50,56,65,75], developing supportive workplace environment and policies [39,45,50,53,57,76], and ensuring that supervisors were supportive and non-punitive advocates for their teams [39,53,55,63,68]. In high-pressure situations, health workers valued experienced, calm, and communicative supervisors who could help them cope with emotional stress [53,55].

*Finding 17. Requiring institutional support* **Health workers required institutional support from their workplace, including mental health, emotional support and guidance. Many workplaces failed to provide this support, and when available, the support often focused on investigating the clinical aspects of the event or criticising health workers, rather than addressing the emotional needs of health workers** (Moderate confidence) [36,38,39,41,45–47,50,53,55,56,58,60,61,64,67,68,73–75,79,84,85].

Health workers reported needing that support after caring for women and babies who had died or experienced complications or deaths, as well as when caring for women from marginalised backgrounds, and when facing conflicts between personal, professional and organisational values [36,39,41,45–47,55,56,60,64,67,73,74,79,85]. Some wanted mental health assistance from professionals who understand the demands of their work [36,38,45,60,61,68,73,85], or who have relevant training [64], while others preferred more informal approaches, such as informal discussions, debriefing sessions, confidential multidisciplinary group debriefings and time off [39,45,46,50,60,78]. Many valued off-site debriefings to provide a neutral space for processing their experiences [39,45,50].

*Finding 18. Building social network support* **Family and friends played a dual role in supporting health workers. Some health workers perceived that family and friends served as valuable support systems when facing workplace challenges, while others avoided or found limited support in their family and friends** (Moderate confidence) [38,39,45,46,51,54–57,60,64,68,69,74,79,80]**.**

Sharing feelings, debriefing, and engaging in social time with family and friends was beneficial, especially when formal debriefing was not available at work. However, for others, personal life issues with family might contribute to burnout among health workers [51]**.** Some health workers preferred to conceal their stress, avoiding the burden of sharing it with family, while others found family support inadequate or unhelpful [55,56,79].

### 3.3. Well-being domains for health workers providing maternal and newborn care

**Fig 3:**
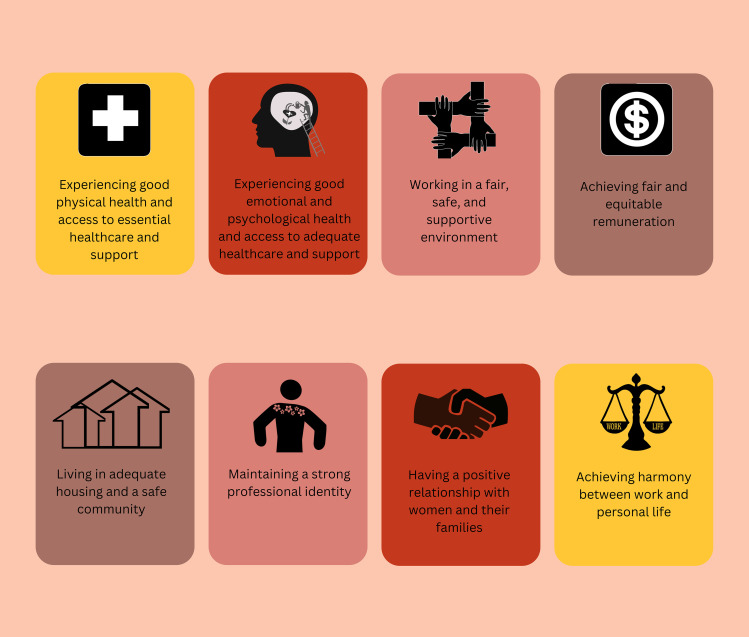
Well-being domains for health workers providing maternal and newborn care.

Fig 3. shows the eight domains of well-being for health workers providing in maternal and newborn care. These domains were developed by mapping the 18 synthesis findings into two existing frameworks of health workers’ well-being: Global health and care workers compact framework [17] and the U.S. Surgeon General’s Framework for Workplace Mental Health and Well-Being [18] and finalised through sense-checking consultations with 18 health workers. Details of each domain can be found in S8 Appendix.

### 3.4. Mapping qualitative synthesis to behaviour change frameworks and potential strategies

Table 3 presents the mapping of synthesis findings to the TDF [34] and COM-B [35] frameworks, and the Behaviour Change Wheel [35] to inform strategies to improve well-being for health workers providing maternal and newborn care. We found that the well-being issues identified in health workers are primarily related to their Automatic and Reflective Motivation, which are psychological processes that energise and direct health workers’ behaviour towards improving or maintaining well-being. Health workers’ motivation is influenced by Physical and Social Opportunities, which are external factors that affect their behaviour towards improving or maintaining well-being. These external factors include organisational, health system and policy factors, such as unhealthy workplace culture, inadequate remuneration, limited opportunities for growth, and insufficient health infrastructure. Out of 18 synthesis findings, only six were related to health workers’ Physical and Psychological Capabilities, which are health workers’ capacity to engage in behaviours that promote or maintain well-being, including their knowledge, skills, and behavioural regulation. This mapping result suggests that improving health workers’ well-being requires strategies that primarily focus on enhancing health workers’ motivation and addressing external factors that influence their well-being, across the organisational, health system, and policy levels. We provided examples of strategies, which were developed through the health workers’ sense-checking consultations, that can be considered to address each of the well-being issues. Each strategy should be discussed, prioritised and tailored to a specific context.

**Table 3:** Mapping qualitative findings to the TDF and COM-B frameworks and BCW.

Findings	QES findings	COM-B and TDF mapping	Actors	Potential intervention type based on BCW	Examples of how implementation strategies could be operationalised, or actual implementation strategies used
1	Valuing the relationship between health workers and women and their families.	**Automatic motivation** (Emotion); **Reflective motivation** (Goals)	Health workers, health facility managers, health workers’/professionals’ associations	Environmental restructuring, Modelling, Enablement, Education	Raise awareness by providing credible information to health facility managers, staff, and women and their families about the significant emotional impact that clinical care can have on health workers. Develop a system of regular, confidential well-being assessments for all health workers and provide resources and support based on the results of the assessments Provide emotional resilience training for health workers, contextually tailored to the setting, to help them regulate their own emotional well-being Identify and train successful health workers who have overcome emotional distress resulting from poor relationships with women and create opportunities for these ambassadors to mentor other health workers. Communicate transparently with women and families the procedures, process, benefits, and risks associated with it to prevent negative impact and blame
2	Experiencing the emotional toll of adverse events.	**Automatic motivation** (Emotion); **Reflective motivation** (Beliefs about capabilities, Social/professional role and identity)	Health workers, health facility managers, health workers’/professionals’ associations	Modelling, Enablement, Education	Develop a ‘safe space’ protocol where health workers can take time out after traumatic events Develop a mentoring programme where experienced staff can share how they have overcome similar challenges Develop resources (e.g., handbooks, online modules) on coping strategies and self-care techniques contextually tailored to the setting. Develop a policy to encourage health workers to take leave after traumatic events Establish a morning conference program to review cases of complications or deaths for learning experience, not blaming Establish a secondary opinion program where health workers are paired with their senior health workers to seek validation and recommendations and build confidence before undertaking major procedures.
3	Needing fair and equitable remuneration.	**Automatic motivation** (Emotion); **Physical opportunity** (Environmental context and resources)	Health workers, health facility managers, health workers’/professionals’ associations, Policymakers	Incentivisation, Environmental restructuring, Enablement, Persuasion	Develop a benefits package that goes beyond basic salary, including professional development opportunities, additional leave for rural workers, transportation and housing support, child education support and health and wellness programmes. Develop a policy of incentives for health workers who have overtime working hours Engage respected healthcare leaders, policymakers, and professional associations to advocate for fair and equitable remuneration
4	Struggling with unhealthy workplace culture.	**Automatic motivation** (Emotion); **Physical opportunity** (Environmental context and resources)	Health workers, health facility managers, health workers’/professionals’ associations	Environmental restructuring, Enablement, Modelling	Train people in managerial positions on the importance of a healthy workplace culture and how to build that culture Establishment of clear communication channels, including feedback when conflicts arise Create a ‘Culture Committee’ with representatives from all levels to drive positive change. Implement a system of regular, confidential wellbeing assessments for all staff Identify a champion for healthy workplace behaviour to encourage respectful work relationships.
5	Dealing with heavy workloads.	**Automatic motivation** (Emotion); **Physical capability** (Skills); **Psychological capability** (Knowledge); **Physical opportunity** (Environmental context and resources)	Health workers, health facility managers, health workers’/professionals’ associations	Environmental restructuring, Training, Enablement, Education, Restriction	Provide training for individuals in managerial positions on better workload management, contextually tailored to the setting. Offer clinical training for health workers to enhance their clinical skills. Raise awareness about how poor workload management and heavy workloads affect the physical and emotional well-being of health workers. Establish a system that allows health workers to access mental and physical health support. Develop a policy or regulation that requires health workers to work a healthy duration of working hours and are entitled to annual leave
6	Finding opportunities for professional growth.	**Automatic motivation** (Reinforcement); **Reflective motivation** (Goals, Social/professional role and identity); **Physical opportunity** (Environmental context and resources)	Health facility managers, Policymakers	Environmental restructuring, Enablement, Incentivisation	Set up a resource centre or e-learning where health workers can learn at their own pace Integrate professional development into staffing plans Introduce a system that allows health workers to request roster adjustments to accommodate training commitments. Schedule learning blocks into shift patterns. Use reminders and calendars to reinforce development time. Include opportunity for training/education as part of the benefit package Establish a knowledge-sharing session among health workers to offer an opportunity for health workers to share their work and achievements
7	Working with an inadequate infrastructure.	**Automatic motivation** (Emotion); **Physical opportunity** (Environmental context and resources)	Health facility managers, health workers’/professionals’ associations, Policymakers	Environmental restructuring, Enablement, Persuasion	Develop a prioritised list of improvements and advocate to policymakers and health facility managers to ensure continuous basic supply Offer counselling services to help midwives deal with frustration and work-related stress. Provision of insurance to cover occupational injuries
8	Facing socio-gendered challenges in rural areas.	**Reflective motivation** (Belief about consequences); **Physical opportunity** (Environmental context and resources); **Social opportunity** (Social influences)	Health facility managers, health workers’/professionals’ associations, Policymakers	Environmental restructuring, Enablement, Education, Persuasion	Conduct a comprehensive assessment of living conditions and infrastructure in rural postings and improve them based on the assessments Establish a mentorship programme pairing new rural health workers with experienced colleagues Implement a confidential reporting system for gender-based discrimination or harassment and provide access to counselling services Develop comprehensive information packages, contextually tailored to the setting, about the realities of rural postings, including both opportunities and challenges Develop and enforce clear policies against gender-based discrimination and harassment Advocate for policymakers on better living conditions and work protection for female health workers going to rural postings
9	Encountering negative or inaccurate media portrayals.	**Reflective motivation** (Belief about consequences, Social/professional role and identity); **Physical opportunity** (Environmental context and resources)	Media, Health facility managers, health workers’/professionals’ associations, Health workers	Environmental restructuring, Enablement, Education	Develop clear policies and procedures for how the health facility will support staff in the face of media scrutiny Provide training for health workers on how to engage with media within the bounds of professional confidentiality Establish a dedicated support team within the health facility to assist health workers affected by negative media coverage and provide access to counselling services for health workers and their families. Provide training for health workers on how to navigate the emotional impact of media coverage on their families and themselves Develop a safe place for colleagues to support each other
10	Fearing mental health stigma.	**Automatic motivation** (Emotion); **Physical opportunity** (Environmental context and resources); **Social opportunity** (Social influences)	Health facility managers, health workers’/professionals’ associations	Environmental restructuring, Enablement, Modelling	Create a campaign within the health facility to normalise help-seeking behaviour, for instance, engage with respected healthcare leaders to openly discuss their own experiences with mental health support or create informational materials highlighting the prevalence of mental health issues among health workers and the benefits of seeking support Create dedicated, private spaces within healthcare facilities for mental health consultations Provide training sessions on how to access mental health support services, including practical steps and what to expect Create anonymous online forums where health workers can share experiences and offer mutual support Identify a mental health champion within the health facility
11	Experiencing pressure from litigation and investigation.	**Automatic motivation** (Emotion); **Reflective motivation** (Belief about consequences); **Physical opportunity** (Environmental context and resources)	Health facility managers, health workers’/professionals’ associations	Environmental restructuring, Enablement, Modelling, Education	Create clear policies on institutional support during lawsuits and investigations. Develop comprehensive information packages explaining audit, lawsuit, and investigation processes Engage respected healthcare leaders to openly discuss their experiences with audits, lawsuits, or investigations Develop a feedback mechanism for health workers to report concerns about the fairness of processes Establish a campaign on how to navigate medical lawsuits and encourage positive peer support
12	Finding strength in professional identity.	**Reflective motivation** (Belief about consequences, Social/professional role and identity); **Physical opportunity** (Environmental context and resources)	Health workers, Health facility managers, health workers’/professionals’ associations	Environmental restructuring, Enablement, Education	Review and revise facility policies to better align with professional values and increase autonomy where possible. Implement a shared decision-making model for policy development, involving health workers at all levels. Create a campaign to promote respectful and mindful collaboration across professions
13	Valuing spirituality.	**Reflective motivation** (Optimism); **Psychological capability** (Behavioural regulation)	Health workers, Health facility managers, health workers’/professionals’ associations	Enablement	Develop policies that respect and accommodate diverse spiritual beliefs and practices Create multi-faith prayer or meditation rooms in healthcare facilities
14	Achieving work-life harmony.	**Reflective motivation** (Social/professional role and identity); **Physical capability** (Skills); **Psychological capability** (Behavioural regulation); **Physical opportunity** (Environmental context and resources)	Health workers, Health facility managers, health workers’/professionals’ associations	Training, Enablement, Environmental restructuring	Develop e-learning modules on effective strategies for achieving work-life harmony Create quiet spaces for relaxation and personal time within the workplace Implement a system for staff to anonymously submit work-life balance challenges for collective problem-solving Remove system barriers (e.g., staff shortages, heavy workloads) to ensure health workers can achieve work-life harmony
15	Receiving reliable peer support.	**Automatic motivation** (Emotion); **Social opportunity** (Social influences)	Health workers, Health facility managers, health workers’/professionals’ associations	Enablement, Environmental restructuring	Develop a confidential online platform for health workers to connect and support each other Develop guidelines for supportive communication and behaviour in the workplace Engage respected healthcare leaders to openly discuss the importance of peer support
16	Accessing adequate mentoring, supervision, and leadership.	**Physical capability** (Skills); **Psychological capability** (Knowledge); **Physical opportunity** (Environmental context and resources); **Social opportunity** (Social influences)	Health workers, Health facility managers, health workers’/professionals’ associations	Enablement, Environmental restructuring, Training, Modelling	Develop and implement a ‘Supportive Leadership’ training programme for all supervisors, including how to negotiate with upper management and staff and to work collaboratively Train supervisors in providing emotional and psychological support Establish a structured mentorship programme pairing experienced staff with junior colleagues
17	Requiring institutional support.	**Physical opportunity** (Environmental context and resources); **Social opportunity** (Social influences)	Health workers, Health facility managers, health workers’/professionals’ associations	Enablement, Environmental restructuring, Modelling	Establish a ‘no-blame’ culture for discussing and learning from adverse events Identify a champion to promote mental health support and seeking behaviours Develop a system of regular, confidential well-being assessments for all health workers and provide resources and support based on the results of the assessments
18	Building social network support.	**Social opportunity** (Social influences)	Health workers, Health facility managers	Enablement	Improve workload management to allow health workers time with family. Offer a lottery system to give staff recreational passes with families

## 4. Discussion

Our QES synthesised perceptions and experiences of well-being among health workers who provide routine maternal and newborn care globally. The QES findings were used to develop domains of well-being for these health workers and informed the development of potential implementation strategies to improve their well-being. Many themes are common to the factors influencing the well-being of all health workers and echo the broader health workforce literature and policy discourse. For instance, some of the most common burnout drivers among nurses in general were workload, the lack of supportive colleagues, role balancing at home and at work, limited growth opportunities, and moral distress [87]. However, some are specific to health workers providing maternal and newborn care. In particular, we identified that these health workers valued their relationship with women, newborns and their families, but poor pregnancy and birth outcomes significantly affected their mental and physical health. Working conditions, including inadequate and inequitable remuneration, unhealthy workplace culture, heavy workloads, staff shortages, inadequate skills, poor workload management, a lack of growth opportunities, and insufficient infrastructure, undermined their well-being. Social, gender, and legal factors affected health workers, such as poor and unsafe living conditions in rural areas, gender dynamics hindering career progression for female health workers, inaccurate media portrayals on litigation/disputed cases, mental health stigma, and a lack of support during clinical audits or investigations. However, health workers valued various forms of support to enhance their well-being, including maintaining professional identities, engaging in spiritual beliefs and practices, achieving work-life harmony, and receiving support from peers, mentors, supervisors, family and friends.

The importance of health worker well-being has been highlighted in several guidelines and reports, which provide recommendations to support mental health at work [88], occupational health and safety [89], and mental health during outbreaks and pandemics [90]. While not specifically focused on health workers providing maternal and newborn care, these guidelines and reports highlight similar well-being issues, including heavy workloads, and poor workload management [88,90] (Finding 5). World Health Organization and International Labour Organization (89) emphasised the need for compensation and access to essential health care for work-related injuries, aligning with our Finding 4. Similarly, the importance of training managers to support health workers’ mental health [88], corresponds to our finding on the crucial role of supervisors or managers in managing workplace stress.

Our QES findings were used to develop eight domains of well-being (see Fig 2), expanding and complementing the Global Health and Care Workers Compact Framework [17]. Our domains provided more detail in certain areas. For example, we distinguish between access to physical and mental health, recognising that they may involve different systems and challenges. We also recognised work-life harmony as a distinct domain, rather than subsuming it under general well-being. Our domains add crucial dimensions not explicitly covered in the Global Health and Care Workers Compact Framework [17], which are the significance of professional self-concept in overall well-being and the impact of health workers’ interactions with women and parents on health workers’ well-being. Moreover, our domains extend well-being domains beyond the workplace by including personal life and community factors, such as community safety, adequate housing and the importance of balancing professional responsibilities with personal life. Therefore, our domains provide a more nuanced and context-specific approach to understand and promote the well-being of health workers providing maternal and newborn care, while still aligning with the core principles of the Global Health and Care Workers Compact Framework.

The TDF and COM-B mapping in Table 3 show that structural and deep-seated external factors, including organisational, health system and policy factors, primarily influence the well-being of health workers providing maternal and newborn care. This finding was consistent with insights gathered from our sense-checking consultations with health workers, who indicated that these factors had a significant impact on their well-being. Through the consultations, health workers emphasised the critical role of strong leadership from supervisors and managers in addressing workplace issues that impact their physical and mental health, including workload management and the provision of mental health support. Health workers in LMICs identified systemic issues like staff shortages, heavy workloads, and inadequate infrastructure as primary sources of distress, arguing that health system improvements would be more effective than mental health support alone. In contrast, health workers in high-income countries highlighted the lack of funding for long-term mental health support and the absence of policies protecting working hours and leave. More broadly, addressing these deeper challenges requires sustained and long-term investments in health workers’ quality education, employment, management and decent working conditions [91].

### 4.1. Strengths and limitations

Our QES aimed to synthesise perceptions and experiences of global health workers, but most sampled papers were from high-income countries, which may affect the transferability of these findings to LMICs. We attempted to sample more records from LMICs, but none met the sampling criteria due to thin data. This limitation highlights that more high quality qualitative research is needed to explore the well-being of health workers providing maternal and newborn care in LMICs. The scope of our QES meant that we did not include care provided in intensive care units and in humanitarian and pandemic or outbreak settings. Therefore, our syntheses and domains may not reflect the needs of health workers working in those settings.

Despite these limitations, to the best of our knowledge, this is the first synthesis aimed at understanding the perceptions and experiences of health workers providing maternal and newborn care regarding their well-being and developing domains of well-being. To enhance the relevance of our findings, we conducted sense-checking consultations with health workers from different geographies. While the relationship between well-being and quality of care was not directly assessed in this review, existing literature suggests that compromised well-being might negatively affect the provision of respectful maternal and newborn care [12–14]. The WHO Compendium on Respectful Maternal and Newborn Care [15] has also identified the well-being of health workers as one of the key areas of intervention to end mistreatment and achieve respectful maternal and newborn care. Therefore, the proposed behaviour change interventions to address well-being should be considered to optimise the provision of respectful maternal and newborn care through improving health workers’ well-being.

## 5. Conclusions

We identified eight key domains that contribute to the well-being of health workers involved in maternal and newborn care. These domains indicate that the well-being of health workers is influenced by a variety of factors, including personal, interpersonal, institutional, systemic, policy-related, and cultural elements. Our behaviour change frameworks can help inform strategies aimed at enhancing the well-being of health workers and can be adapted and tailored to specific contexts.

## Supporting information

S1 Appendix Enhancing Transparency in Reporting the Synthesis of Qualitative Research: (ENTREQ) reporting checklist. (DOCX)

S2 Appendix Search strategies. (DOCX)

S3 Appendix Sampling process. (DOCX)

S4 Appendix CochrAne qualitative Methodological LimitatiOns Tool (CAMELOT) Critical Appraisal. (DOCX)

S5 Appendix Characteristics of sampled papers. (DOCX)

S6 Appendix Characteristics of papers awaiting classification. (DOCX)

S7 Appendix GRADE-CERQual Evidence Profile. (DOCX)

S8 Appendix Well-being domains for health workers providing maternal and newborn care. (DOCX)
