# Supplementary material for: Well-being of health workers providing maternal and newborn care: A qualitative evidence synthesis
Source: PLOS Glob Public Health. 2026 Feb 11;6(2):e0005522. doi: 10.1371/journal.pgph.0005522 (PMC12893595; doi:10.1371/journal.pgph.0005522)
Supplement: S2 Appendix — (DOCX) [file pgph.0005522.s002.docx]

# S2 Appendix. Search strategies

## CINAHL

((MH "Nurse Midwifery") OR (MH "Maternal-Child Nursing") OR (MH "Perinatal Nursing+") OR (MH "Perinatal Nurses+") OR (MH "Midwives+") OR (MM "Obstetricians") OR (MM "Neonatologists") OR (TI midwifery OR AB midwifery) OR (TI midwives OR AB midwives) OR (TI obstetricians OR AB obstetricians) OR (TI neonatologists OR AB neonatologists) OR (((TI doctors OR AB doctors) OR (TI nurses OR AB nurses) OR (TI nursing OR AB nursing) OR (TI workers OR AB workers) OR (TI personnel OR AB personnel) OR (TI professionals OR AB professionals) OR (TI practitioners OR AB practitioners) OR (TI staff OR AB staff)) N10 ((TI maternal OR AB maternal) OR (TI maternity OR AB maternity) OR (TI newborn OR AB newborn) OR (TI NICU OR AB NICU) OR (TI antenatal OR AB antenatal) OR (TI pregnancy OR AB pregnancy) OR (TI postnatal OR AB postnatal) OR (TI intrapartum OR AB intrapartum) OR (TI childbirth OR AB childbirth) OR (TI birth OR AB birth) OR (TI "obstetric delivery" OR AB "obstetric delivery")))) AND ((MH "Stress, Occupational+") OR (MH "Hardiness") OR (MH "Job Satisfaction+") OR (MH "Workload") OR (MH "Work-Life Balance") OR (MH "Job Security") OR (MH "Emotional Exhaustion") OR ((MH Aggression) AND (MH "Health Personnel+")) OR (TI wellbeing OR AB wellbeing) OR (TI well-being OR AB well-being) OR (TI burnout OR AB burnout) OR (TI resilience OR AB resilience) OR (TI stress OR AB stress) OR (TI emotion* OR AB emotion* ) OR (TI self-esteem OR AB self-esteem) OR (TI coping OR AB coping) OR (TI "quality of life" OR AB "quality of life") OR ((TI job OR AB job) N5 (TI satisf* OR AB satisf*)) OR (TI retention OR AB retention) OR (TI aggressi* OR AB aggressi*) OR (TI harass* OR AB harass*) OR (TI bullying OR AB bullying) OR (TI bullied OR AB bullied)) AND ((MH "Qualitative Studies+") OR (MH "Interviews+") OR (MH "Focus Groups") OR (TI qualitative OR AB qualitative) OR (TI "focus* group*" OR AB "focus* group*") OR (TI interview* OR AB interview*) OR ((TI participatory OR AB participatory) N3 (TI research OR AB research)) OR ((TI thematic OR AB thematic) N2 (TI analysis OR AB analysis)) OR (TI themes OR AB themes) OR (TI "mixed method#" OR AB "mixed method#") OR (TI phenomenolog* OR AB phenomenolog*) OR (TI ethnograph* OR AB ethnograph*))

## MIDIRS

((midwifery or midwives or obstetricians or neonatologists or ((doctors or nurses or nursing or workers or personnel or professionals or practitioners or staff) adj10 (maternal or maternity or newborn or NICU or antenatal or pregnancy or postnatal or intrapartum or childbirth or birth or obstetric delivery))) and (wellbeing or well-being or burnout or resilience or stress or emotion* or self-esteem or coping or "quality of life" or (job adj5 satisf*) or retention or aggressi* or harass* or bullying or bullied) and (qualitative or focus* group* or interview* or (participatory adj3 research) or (thematic adj2 analysis) or themes or mixed method? or phenomenolog* or ethnograph*)).ti,ab.

## MEDLINE

(Midwifery/ or Nurse Midwives/ or exp Maternal-Child Nursing/ or Obstetric Nursing/ or Obstetricians/ or Neonatologists/ or (midwifery or midwives or obstetricians or neonatologists or ((doctors or nurses or nursing or workers or personnel or professionals or practitioners or staff) adj10 (maternal or maternity or newborn or NICU or antenatal or pregnancy or postnatal or intrapartum or childbirth or birth or obstetric delivery))).ti,ab,kf.) and (exp Stress, Psychological/ or Resilience, Psychological/ or Job Satisfaction/ or Workload/ or Work-Life Balance/ or Job Security/ or Emotions/ or (exp Aggression/ and exp Health Personnel/) or (wellbeing or well-being or burnout or resilience or stress or emotion* or self-esteem or coping or "quality of life" or (job adj5 satisf*) or retention or aggressi* or harass* or bullying or bullied).ti,ab,kf.) and (Qualitative Research/ or Grounded Theory/ or Interviews as Topic/ or Focus Groups/ or (qualitative or focus* group* or interview* or (participatory adj3 research) or (thematic adj2 analysis) or themes or mixed method? or phenomenolog* or ethnograph*).ti,ab,kf.)
