# Supplementary material for: Well-being of health workers providing maternal and newborn care: A qualitative evidence synthesis
Source: PLOS Glob Public Health. 2026 Feb 11;6(2):e0005522. doi: 10.1371/journal.pgph.0005522 (PMC12893595; doi:10.1371/journal.pgph.0005522)
Supplement: S3 Appendix — (DOCX) [file pgph.0005522.s003.docx]

# S3 Appendix. Sampling strategies


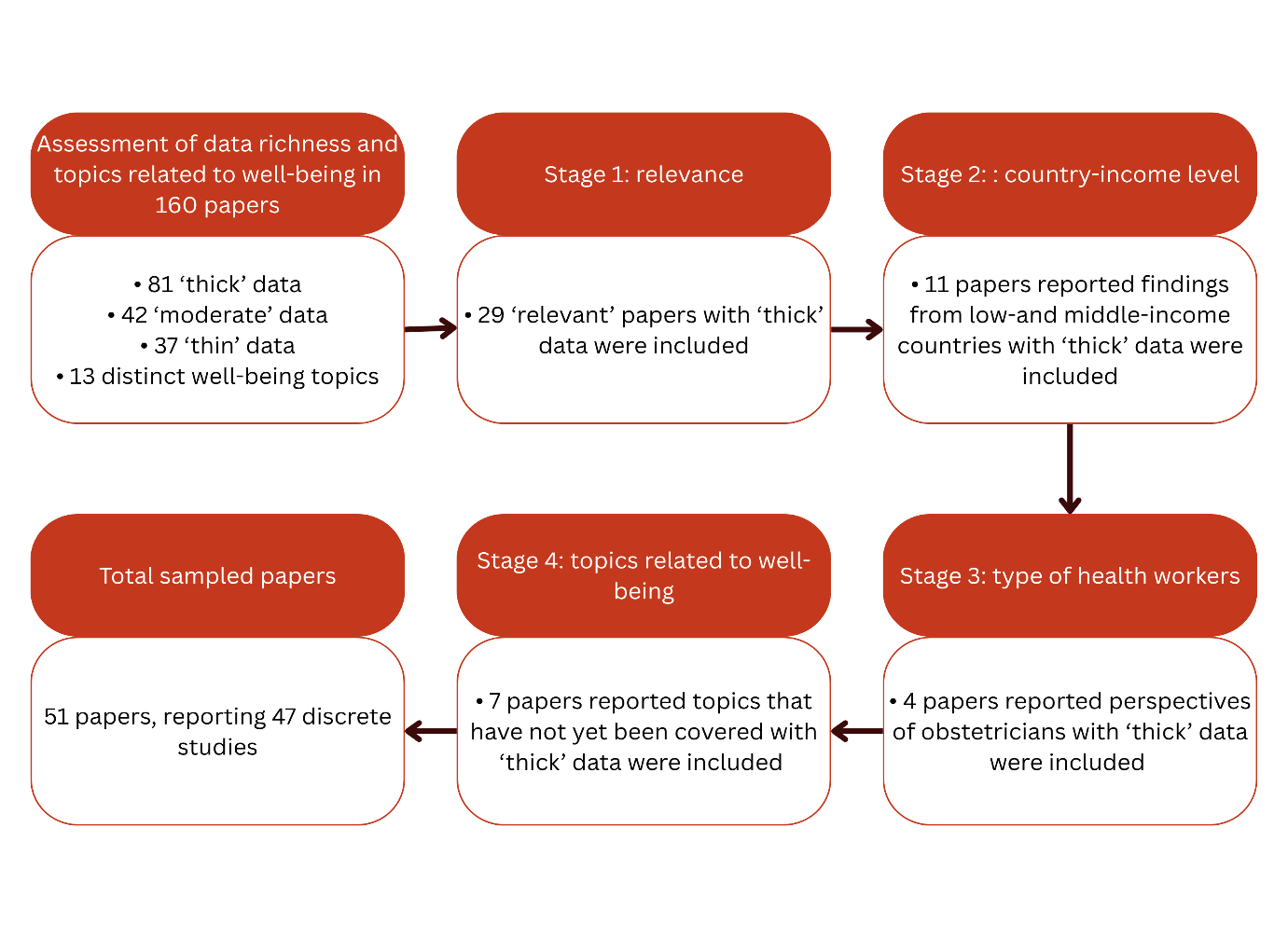


First, we assessed the data richness and topics related to well-being in the 160 papers. We assessed data richness of the papers into ‘thick’, ‘moderate’ and ‘thin’ and considered data to be ‘thick’, if most of the results section discussed well-being. This assessment of data richness was used in combination with other sampling criteria. We also identified 13 distinct well-being topics discussed across the 160 papers, such as social/work dynamics, care schemes, physical/geographical work environments, and complex case situations. In Stage 1 of the sampling process, we assessed the relevance of the 160 papers. Papers were considered ‘relevant’ when the primary objective was to explore health workers’ well-being and presented findings on the topic (45 papers were classified as ‘relevant’). Papers were considered ‘partially relevant’ if health workers’ well-being was not a primary aim of the study but was discussed in the findings (115 papers were classified as ‘partially relevant’). We evaluated the data richness of the 45 ‘relevant’ papers; of these, 29 papers had ‘thick’ data and were included. In Stage 2, we evaluated the country income levels of the 29 papers sampled in Stage 1. Since 20 of 29 papers already included from the ‘relevant and thick data’ stage were from high-income countries, we added all 11 papers from low- and middle-income countries (LMICs) that were partially relevant and had thick data, to ensure variation across different settings (40 papers sampled in total at this stage). In Stage 3, we assessed the types of health workers addressed in the 40 sampled papers to ensure diverse perspectives within the population of interest. As most included papers reported on midwives' perspectives, we added all four papers that presented the perspectives of obstetricians with partially relevant and rich data (44 papers sampled in total at this stage). Finally, we evaluated topics related to well-being in the 44 sampled papers and added seven papers to ensure all topics were represented, giving a total of 51 papers (reporting 47 discrete studies). This assessment process involved two reviewers (AH, NM), and the stages of sampling and decisions were iteratively discussed with the review team.
