# Supplementary material for: Well-being of health workers providing maternal and newborn care: A qualitative evidence synthesis
Source: PLOS Glob Public Health. 2026 Feb 11;6(2):e0005522. doi: 10.1371/journal.pgph.0005522 (PMC12893595; doi:10.1371/journal.pgph.0005522)
Supplement: S4 Appendix — (DOCX) [file pgph.0005522.s004.docx]

## S4 Appendix. CochrAne qualitative Methodological LimitatiOns Tool (CAMELOT) Critical Appraisal

| **Nu** | **Authors** | **Fit between Research design domains and Research aim & question** | **Fit between Research design domains and Stakeholders** | **Fit between Research design domains and Researchers** | **Fit between Research design domains and Context** | **Fit between Research design domains and Research conduct domains** | **Fit between Research design domains and Research aim & question** | **Fit between Research conduct domains and Stakeholders** | **Fit between Research conduct domains and Researchers** | **Fit between Research conduct domains and Context** | **Overall assessment of concerns** |
| --- | --- | --- | --- | --- | --- | --- | --- | --- | --- | --- | --- |
| 1 | Abraham (2020) | No or minimal concerns | No or minimal concerns | Serious concerns (unclear) | No or minimal concerns | Moderate concerns | Moderate concerns | No or minimal concerns | Serious concerns (unclear) | No or minimal concerns | Moderate concerns |
|  |  | NA | NA | Researchers' backgrounds, roles, and reflexivity not reported. | NA | Unclear who and on how many researchers conducted the participant recruitment and selection, data collection and data analysis. | Data analysis process unclear; no information on managing emotional risks for sensitive topics; equity/inclusion/diversity not addressed. | NA | Researchers' backgrounds, roles, and reflexivity not reported. | NA | Researchers' backgrounds, roles, and reflexivity unreported; data analysis process unclear; no information on managing emotional risks for sensitive topics; equity/inclusion/diversity not addressed. |
| 2 | Adatara (2021) | No or minimal concerns | No or minimal concerns | Moderate concerns | No or minimal concerns | Minor concerns | No or minimal concerns | No or minimal concerns | Moderate concerns | No or minimal concerns | Minor concerns |
|  |  | NA | NA | Researchers' contributions reported, but backgrounds and their impact on research design/implementation not disclosed. | NA | Influence of researchers' backgrounds on their relationship with participants and data interpretation not addressed; equity/inclusion/diversity not addressed in recruitment strategy. | NA | NA | Researchers' contributions reported, but backgrounds and their impact on research design/implementation not disclosed. | NA | Researchers' contributions reported, but backgrounds and their impact on research design/implementation not disclosed; influence of researchers' backgrounds on their relationship with participants and data interpretation not addressed; equity/inclusion/diversity not addressed in recruitment strategy. |
| 3 | Allam (2023) | No or minimal concerns | No or minimal concerns | Serious concerns (unclear) | No or minimal concerns | Serious concerns | Serious concerns | No or minimal concerns | Serious concerns (unclear) | No or minimal concerns | Serious concerns |
|  |  | NA | NA | Researchers' backgrounds, roles, and reflexivity not reported. | NA | Ethical considerations not reported, data analysis and collection processes unclear; researchers conducting data collection and analysis not specified; recruitment criteria and process unclear. | Ethical considerations not reported, data analysis and collection processes unclear; researchers conducting data collection and analysis not specified; recruitment criteria and process unclear. | NA | Researchers' backgrounds, roles, and reflexivity not reported. | NA | Researchers' backgrounds, roles, and reflexivity not reported; ethical considerations not reported, data analysis and collection processes unclear; researchers conducting data collection and analysis not specified; recruitment criteria and process unclear |
| 4 | André (2019) | No or minimal concerns | No or minimal concerns | Minor concerns | Minor concerns | Moderate concerns | Minor concerns | No or minimal concerns | Minor concerns | Minor concerns | Minor concerns |
|  |  | NA | NA | Impact of researchers' backgrounds on study implementation and participant relationships not addressed. | Insufficient context provided on midwives' demographics and available support | Unclear recruitment process may exclude potential participants; lack of theoretical support for findings interpretation; no consideration of diversity. | No information on managing emotional risks for sensitive topics. | NA | Impact of researchers' backgrounds on study implementation and participant relationships not addressed. | Insufficient context provided on midwives' demographics and available support | Impact of researchers' backgrounds on study implementation and participant relationships not addressed; Insufficient context provided on midwives' demographics and available support; unclear recruitment process may exclude potential participants; lack of theoretical support for findings interpretation; no consideration of diversity; no information on managing emotional risks for sensitive topics. |
| 5 | Arbour (2020) | No or minimal concerns | Minor concerns | Serious concerns (unclear) | Serious concerns (unclear) | Moderate concerns | No or minimal concerns | Minor concerns | Serious concerns (unclear) | Serious concerns (unclear) | Moderate concerns |
|  |  | NA | Stakeholders involved, conflicts of interest not addressed. | Researchers' backgrounds, roles, and reflexivity not reported. | No information on context | Unclear who and how many researchers analysed data; lack of diverse perspectives despite online survey use; no theoretical framework for interpreting findings. | NA | Stakeholders involved, conflicts of interest not addressed. | Researchers' backgrounds, roles, and reflexivity not reported. | No information on context | Stakeholders involved, conflicts of interest not addressed; researchers' backgrounds, roles, and reflexivity not reported; no information on context; lack of diverse perspectives despite online survey use; no theoretical framework for interpreting findings. |
| 6 | Beck (2012) | No or minimal concerns | Minor concerns | Moderate concerns | Serious concerns (unclear) | Moderate concerns | No or minimal concerns | Moderate concerns | Moderate concerns | Serious concerns (unclear) | Moderate concerns |
|  |  | NA | Stakeholders involved, conflicts of interest not addressed. | Research assistants' backgrounds reported, but main researchers' backgrounds and their impact on study design not disclosed. | No information on context | Unclear sample development justification led to potential participant exclusion; failure to include diverse perceptions and experiences; lack of theoretical framework for findings interpretation. | NA | Stakeholders involved in sampling but justification for sample creation unclear. | Research assistants' backgrounds reported, but main researchers' backgrounds and their impact on study design not disclosed. | No information on context | Stakeholders involved, conflicts of interest not addressed; main researchers' backgrounds and their impact on study design not disclosed; unclear sample development justification; failure to include diverse perceptions and experiences; lack of theoretical framework for findings interpretation. |
| 7 | Beck (2016) | No or minimal concerns | Minor concerns | Serious concerns (unclear) | Serious concerns (unclear) | Minor concerns | No or minimal concerns | Moderate concerns | Serious concerns (unclear) | Serious concerns (unclear) | Moderate concerns |
|  |  | NA | Stakeholders involved, conflicts of interest not addressed. | Researchers' backgrounds, roles, and reflexivity not reported. | No information on context | Unclear sample development justification led to potential participant exclusion; failure to include diverse perceptions and experiences. | NA | Stakeholders involved in sampling but justification for sample creation unclear. | Researchers' backgrounds, roles, and reflexivity not reported. | No information on context | Stakeholders involved, conflicts of interest not addressed; main researchers' backgrounds and their impact on study design not disclosed; unclear sample development justification; failure to include diverse perceptions and experiences. |
| 8 | Beck (2020) | Moderate concerns | No or minimal concerns | Moderate concerns | Serious concerns (unclear) | Moderate concerns | No or minimal concerns | No or minimal concerns | Moderate concerns | Serious concerns (unclear) | Serious concerns |
|  |  | Lacks diverse perceptions and experience. | NA | No researchers’ reflexivity | No information on context | Impact of main researchers' backgrounds on data interpretation unclear; diversity not considered; no theoretical framework used | NA | NA | No researchers’ reflexivity | No information on context | Impact of main researchers' backgrounds on data interpretation unclear; diversity not considered; no theoretical framework used; no researchers’ reflexivity; no information on context |
| 9 | Becker (2022) | No or minimal concerns | No or minimal concerns | Minor concerns | No or minimal concerns | No or minimal concerns | Minor concerns | No or minimal concerns | Minor concerns | No or minimal concerns | Minor concerns |
|  |  | NA | NA | Insufficient information on researchers' backgrounds and reflexivity | NA | NA | Unclear use of observational data (body language) in analysis; no information on managing emotional risks for sensitive topics. | NA | Insufficient information on researchers' backgrounds and reflexivity | NA | Insufficient information on researchers' backgrounds and reflexivity; unclear use of observational data (body language) in analysis; no information on managing emotional risks for sensitive topics. |
| 10 | Becker (2023) | No or minimal concerns | No or minimal concerns | No or minimal concerns | No or minimal concerns | Minor concerns | No or minimal concerns | No or minimal concerns | No or minimal concerns | No or minimal concerns | No or minimal concerns |
|  |  | NA | NA | NA | NA | Lack of theoretical framework for interpreting findings | NA | NA | NA | NA | Lack of theoretical framework for interpreting findings |
| 11 | Bradley (2015) | No or minimal concerns | Minor concerns | Minor concerns | No or minimal concerns | Minor concerns | Minor concerns | Minor concerns | Minor concerns | No or minimal concerns | Minor concerns |
|  |  | NA | Unclear involvement of funders and partner organisations | No researchers’ reflexivity | NA | Lack of theoretical framework for interpreting findings | No information on managing emotional risks for sensitive topics. | Unclear involvement of funders and partner organisations | No researchers’ reflexivity | NA | Unclear involvement of funders and partner organisations; lack of theoretical framework for interpreting findings; no researchers’ reflexivity; no information on managing emotional risks for sensitive topics. |
| 12 | Bremnes (2018) | Minor concerns | Minor concerns | Serious concerns (unclear) | No or minimal concerns | Serious concerns | No or minimal concerns | Minor concerns | Serious concerns (unclear) | No or minimal concerns | Moderate concerns |
|  |  | No rationale provided for research design | Unclear involvement of funders | Researchers' backgrounds, roles, and reflexivity not reported. | NA | Lack of theoretical framework; unclear recruitment process; lack of ethical considerations in recruitment | NA | Unclear involvement of funders | Researchers' backgrounds, roles, and reflexivity not reported. | NA | No rationale provided for research design; unclear involvement of funders; researchers' backgrounds, roles, and reflexivity not reported; lack of theoretical framework; unclear recruitment process; lack of ethical considerations in recruitment. |
| 13 | Calvert (2015) | No or minimal concerns | No or minimal concerns | Serious concerns (unclear) | Serious concerns (unclear) | Minor concerns | No or minimal concerns | No or minimal concerns | Serious concerns (unclear) | Serious concerns (unclear) | Moderate concerns |
|  |  | NA | NA | Researchers' backgrounds, roles, and reflexivity not reported. | No information on context | Recruitment process unclear; equity not considered | NA | NA | Researchers' backgrounds, roles, and reflexivity not reported. | No information on context | Researchers' backgrounds, roles, and reflexivity not reported; no information on context; recruitment process unclear; equity not considered. |
| 14 | Cankaya (2021) | No or minimal concerns | No or minimal concerns | No or minimal concerns | Serious concerns (unclear) | Moderate concerns | Minor concerns | No or minimal concerns | No or minimal concerns | Serious concerns (unclear) | Moderate concerns |
|  |  | NA | NA | NA | Context not provided; rationale for conducting study in Turkey unclear. | Equity,/diversity/inclusion not considered; concerns about recruitment process; no theoretical framework used | No information on managing emotional risks for sensitive topics. | NA | NA | Context not provided; rationale for conducting study in Turkey unclear. | Context not provided; rationale for conducting study in Turkey unclear; equity,/diversity/inclusion not considered; concerns about recruitment process; no theoretical framework used; no information on managing emotional risks for sensitive topics. |
| 15 | Darling (2020) | No or minimal concerns | No or minimal concerns | No or minimal concerns | No or minimal concerns | No or minimal concerns | No or minimal concerns | No or minimal concerns | No or minimal concerns | No or minimal concerns | No or minimal concerns |
|  |  | NA | NA | NA | NA | NA | NA | NA | NA | NA | NA |
| 16 | Darling (2023) | No or minimal concerns | No or minimal concerns | Serious concerns (unclear) | No or minimal concerns | Minor concerns | No or minimal concerns | No or minimal concerns | Serious concerns (unclear) | No or minimal concerns | Minor concerns |
|  |  | NA | NA | Researchers' backgrounds, roles, and reflexivity not reported. | NA | Data management process unclear regarding participants' privacy and confidentiality | NA | NA | Researchers' backgrounds, roles, and reflexivity not reported. | NA | Data management process unclear regarding participants' privacy and confidentiality; researchers' backgrounds, roles, and reflexivity not reported. |
| 17 | Dartey (2019) | No or minimal concerns | No or minimal concerns | Serious concerns (unclear) | No or minimal concerns | Serious concerns | Minor concerns | No or minimal concerns | Serious concerns (unclear) | No or minimal concerns | Serious concerns |
|  |  | NA | NA | Researchers' backgrounds, roles, and reflexivity not reported. | NA | Data collection topics unclear; minimal explanation of sub-themes; equity/diversity/inclusion not considered in research design | Data collection topics unclear | NA | Researchers' backgrounds, roles, and reflexivity not reported. | NA | Data collection topics unclear; minimal explanation of sub-themes; equity/diversity/inclusion not considered in research design; Researchers' backgrounds, roles, and reflexivity not reported. |
| 18 | Dartey (2020) | No or minimal concerns | No or minimal concerns | Serious concerns (unclear) | No or minimal concerns | Serious concerns | Moderate concerns | No or minimal concerns | Serious concerns (unclear) | No or minimal concerns | Serious concerns |
|  |  | NA | NA | Researchers' backgrounds, roles, and reflexivity not reported. | NA | Unclear recruitment process; equity/diversity/inclusion not considered in research design; no theoretical framework used; findings interpretation relies heavily on quotes. | Unclear recruitment process; unclear inclusion/exclusion criteria; insufficient information on FGD and IDI implementation | NA | Researchers' backgrounds, roles, and reflexivity not reported. | NA | Researchers' backgrounds, roles, and reflexivity not reported; Unclear recruitment process; equity/diversity/inclusion not considered in research design; no theoretical framework used; findings interpretation relies heavily on quotes; unclear inclusion/exclusion criteria; insufficient information on FGD and IDI implementation. |
| 19 | Doherty (2022) | Moderate concerns | Serious concerns | Serious concerns (unclear) | No or minimal concerns | Serious concerns | Minor concerns | Serious concerns | Serious concerns | No or minimal concerns | Serious concerns |
|  |  | Study used Participatory Action Research (PAR) methodology, but planning details (e.g., study partners) unclear in research design | Funders' role and study partners unclear | Researchers' backgrounds and reflexivity not reported while it is crucial for power balance in Participatory Action Research (PAR) with co-researchers. | NA | Concerns about the use of convenience sampling; Equity not considered | Equity not considered in recruitment. | Funders' role and study partners unclear | Researchers' backgrounds and reflexivity not reported while it is crucial for power balance in Participatory Action Research (PAR) with co-researchers. | NA | Study used PAR methodology, but planning details unclear in research design, researchers' backgrounds and reflexivity not reported; equity not considered; funders' role and study partners unclear; concerns about the use of convenience sampling. |
| 20 | Doherty (2023) | Moderate concerns | Serious concerns | Serious concerns (unclear) | No or minimal concerns | Serious concerns | Minor concerns | Serious concerns | Serious concerns | No or minimal concerns | Serious concerns |
|  |  | Study used Participatory Action Research (PAR) methodology, but planning details (e.g., study partners) unclear in research design | Funders' role and study partners unclear | Researchers' backgrounds and reflexivity not reported while it is crucial for power balance in Participatory Action Research (PAR) with co-researchers. | NA | Concerns about the use of convenience sampling; Equity not considered | Equity not considered in recruitment. | Funders' role and study partners unclear | Researchers' backgrounds and reflexivity not reported while it is crucial for power balance in Participatory Action Research (PAR) with co-researchers. | NA | Study used PAR methodology, but planning details unclear in research design, researchers' backgrounds and reflexivity not reported; equity not considered; funders' role and study partners unclear; concerns about the use of convenience sampling. |
| 21 | Feeley (2022) | No or minimal concerns | No or minimal concerns | Minor concerns | Serious concerns (unclear) | Minor concerns | No or minimal concerns | No or minimal concerns | Minor concerns | Serious concerns (unclear) | Minor concerns |
|  |  | NA | NA | Lack of researchers’ reflexivity | No information on context | One researcher conducted data analysis, and no evidence of findings consultation with other researchers. | NA | NA | One researcher conducted data analysis, and no evidence of findings consultation with other researchers. | No information on context | No information on context; one researcher conducted data analysis, and no evidence of findings consultation with other researchers. |
| 22 | Fontein-Kuipers (2018) | No or minimal concerns | No or minimal concerns | Serious concerns (unclear) | No or minimal concerns | Serious concerns | No or minimal concerns | No or minimal concerns | Moderate concerns | No or minimal concerns | Serious concerns |
|  |  | NA | NA | Researchers' backgrounds and reflexivity not reported | NA | Unclear who conducted analysis; concerns about researcher's dual role; no theoretical framework used; equity not considered. | NA | NA | Concerns raised about researcher's dual role | NA | Researchers' backgrounds and reflexivity not reported; unclear who conducted analysis; concerns about researcher's dual role; no theoretical framework used; equity not considered. |
| 23 | Geraghty (2019) | No or minimal concerns | No or minimal concerns | Serious concerns (unclear) | No or minimal concerns | Minor concerns | No or minimal concerns | No or minimal concerns | Serious concerns (unclear) | No or minimal concerns | Minor concerns |
|  |  | NA | NA | Researchers' backgrounds and reflexivity not reported | NA | Unclear who conducted data collection and analysis. | NA | NA | Unclear who conducted the data collection and analysis. No reflexivity | NA | Researchers' backgrounds and reflexivity not reported; unclear who conducted data collection and analysis |
| 24 | Gu (2011) | No or minimal concerns | Minor concerns | Moderate concerns | No or minimal concerns | Moderate concerns | No or minimal concerns | Minor concerns | Moderate concerns | Moderate concerns | Moderate concerns |
|  |  | NA | Stakeholder involvement not reported; funders' role not disclosed despite study being funded | Limited information on researchers' roles and backgrounds; reflexivity not addressed | NA | Potential power imbalance due to unclear recruitment process and limited researcher information; no theoretical framework for interpreting/organising findings; participant confidentiality measures not reported | NA | Stakeholder involvement not reported; funders' role not disclosed despite study being funded | Limited information on researchers' roles and backgrounds; reflexivity not addressed; unclear who conducted data analysis | Concerns about power imbalance in the hospital setting due to unclear recruitment process, limited information on researchers, undefined researcher-participant relationships | Stakeholder involvement not reported; funders' role not disclosed despite study being funded; limited information on researchers' roles and backgrounds; reflexivity not addressed; potential power imbalance between researcher and participant |
| 25 | Hajiesmaello (2022) | No or minimal concerns | No or minimal concerns | Minor concerns | No or minimal concerns | Minor concerns | No or minimal concerns | No or minimal concerns | Minor concerns | No or minimal concerns | Minor concerns |
|  |  | NA | NA | Researchers’ reflexivity not reported | NA | No theoretical framework used; sensitive topics addressed without risk mitigation strategies | NA | NA | Researchers’ reflexivity not reported | NA | No theoretical framework used; sensitive topics addressed without risk mitigation strategies; researchers’ reflexivity not reported |
| 26 | Halperin (2011) | No or minimal concerns | Serious concerns (unclear) | Minor concerns | No or minimal concerns | Serious concerns | Moderate concerns | Serious concerns (unclear) | Moderate concerns | Serious concerns | Serious concerns |
|  |  | NA | Stakeholders not reported | Researchers’ reflexivity not reported | NA | Ethical concerns: midwife authors involved in recruitment; lack of reflexivity; unclear interview conduct; confidentiality risks: data collection at participants' unit; managers aware of participation; sampling method may limit diversity of perspectives; no theoretical framework used; equity not considered | Data collection location and managers' awareness of participation raise concerns about confidentiality and data quality | Stakeholders not reported | Ethical concerns: midwife authors involved in recruitment; lack of reflexivity; unclear interview conduct; | Data collection location and managers' awareness of participation raise concerns about confidentiality and data quality | Ethical concerns: midwife authors involved in recruitment; lack of reflexivity; unclear interview conduct; confidentiality risks: data collection at participants' unit; managers aware of participation; sampling method may limit diversity of perspectives; no theoretical framework used; equity not considered; researchers’ reflexivity and stakeholders not reported |
| 27 | Holly (2019) | No or minimal concerns | Minor concerns | Minor concerns | No or minimal concerns | Serious concerns | Moderate concerns | Minor concerns | Moderate concerns | No or minimal concerns | Serious concerns |
|  |  | NA | NHS role in study unclear | No reflexivity provided. | NA | No researcher reflexivity provided; senior midwives' presence may affect focus group dynamics; confidentiality risks due to recruitment through the Head of Midwifery, and power that might influence the participation. | Senior midwife's presence in focus group may impact openness; unclear if participants knew them, potentially affecting responses. | NHS role in study unclear | Senior midwife's presence in focus group may impact openness; unclear if participants knew them, potentially affecting responses; senior midwives' presence may affect focus group dynamics. | NA | No researcher reflexivity provided; senior midwives' presence may affect focus group dynamics; confidentiality risks due to recruitment through the Head of Midwifery, and power that might influence the participation; no reflexivity provided; NHS role in study unclear. |
| 28 | Hunter (2014) | No or minimal concerns | No or minimal concerns | Minor concerns | No or minimal concerns | Moderate concerns | Minor concerns | No or minimal concerns | Minor concerns | No or minimal concerns | Minor concerns |
|  |  | NA | NA | No reflexivity provided; concerns about authors' relationship with expert panel members | NA | No reflexivity provided; concerns about authors' relationship with expert panel members; unclear panel development process; no theoretical framework used | Unclear recruitment criteria | NA | No reflexivity provided; concerns about authors' relationship with expert panel members; unclear panel development process. | NA | No reflexivity provided; concerns about authors' relationship with expert panel members; unclear panel development process; no theoretical framework used; unclear recruitment criteria |
| 29 | Ismaila (2023) | Serious concerns | No or minimal concerns | Moderate concerns | Minor concerns | Serious concerns | Serious concerns | No or minimal concerns | Minor concerns | Minor concerns | Serious concerns |
|  |  | Grounded Theory methodology used without theoretical sampling or apparent constant comparison analysis | NA | Reflexivity not discussed; only first author's background provided | Brief information on context | Grounded Theory methodology used without theoretical sampling or apparent constant comparison analysis; participant approach/recruitment process unclear | Grounded Theory methodology used without theoretical sampling or apparent constant comparison analysis | NA | Reflexivity not discussed; unclear who conducted recruitment and how it was conducted. | Brief information on context | Grounded Theory methodology used without theoretical sampling or apparent constant comparison analysis; participant approach/recruitment process unclear; reflexivity not discussed; brief information on context |
| 30 | Jaffre (2021) | Minor concerns | No or minimal concerns | Minor concerns | No or minimal concerns | Moderate concerns | No or minimal concerns | No or minimal concerns | Minor concerns | No or minimal concerns | Minor concerns |
|  |  | Methodology unclear- appears qualitative but lacks clear methodological grounding. | NA | Researchers’ reflexivity not reported | NA | Unclear methodology and data analysis method - appears qualitative but lacks clear grounding; intentional exclusion of male midwives | NA | NA | Researchers’ reflexivity not reported | NA | Lacks a clear grounding in methodology and data analysis methods; researchers’ reflexivity not reported; intentional exclusion of male midwives |
| 31 | Kave (2023) | No or minimal concerns | Minor concerns | Serious concerns (unclear) | No or minimal concerns | Serious concerns | No or minimal concerns | Minor concerns | Serious concerns (unclear) | Minor concerns | Serious concerns |
|  |  | NA | Funder's roles not reported | Researchers' backgrounds and reflexivity not reported | NA | Short interview duration - possibly due to researchers' skills or topic sensitivity; concerns about managing participants' emotional risks in sensitive research conducted in high perinatal loss district. | NA | Funder's roles not reported | Researchers' backgrounds and reflexivity not reported | Concerns about managing participants' emotional risks in sensitive research conducted in high perinatal loss district. | Short interview duration; concerns about managing participants' emotional risks in sensitive research conducted in high perinatal loss district; researchers' backgrounds and reflexivity not reported; funder's roles not reported |
| 32 | Lawrence (2024) | No or minimal concerns | No or minimal concerns | Minor concerns | No or minimal concerns | Moderate concerns | Minor concerns | No or minimal concerns | Minor concerns | No or minimal concerns | Minor concerns |
|  |  | NA | NA | Only one author's background provided - no information on two researchers who conducted data analysis. | NA | Questionable use of focus group discussions for sensitive topic; specific methodology and theoretical framework not reported | Questionable use of focus group discussions for sensitive topic | NA | Only one author's background provided - no information on two researchers who conducted data analysis. | NA | Questionable use of focus group discussions for sensitive topic; specific methodology and theoretical framework not reported; only one author's background provided. |
| 33 | Lawton (2016) | No or minimal concerns | No or minimal concerns | Serious concerns (unclear) | No or minimal concerns | Moderate concerns | Serious concerns | No or minimal concerns | Serious concerns (unclear) | No or minimal concerns | Serious concerns |
|  |  | NA | NA | Researchers' backgrounds, roles and reflexivity not reported | NA | Key concerns:  participants' confidentiality. unclear researcher-participant relationships,  vague data analysis process, potential participant selection bias. possible influence of senior midwife on recruitment/data collection, despite off-site interviews, unclear who conducted data analysis. | Due to self-selection recruitment, it raises a concern on whether recruited participants were the ones who could provide answers to research questions.  Unclear who conducted data collection and analysis. No information on who conducted these two processes. | NA | Researchers' backgrounds, roles and reflexivity not reported | NA | Key concerns:  participants' confidentiality. unclear researcher-participant relationships,  vague data analysis process, potential participant selection bias. possible influence of senior midwife on recruitment/data collection, despite off-site interviews, unclear who conducted data analysis. |
| 34 | Long (2013) | No or minimal concerns | Minor concerns | Minor concerns | No or minimal concerns | Moderate concerns | No or minimal concerns | Minor concerns | Minor concerns | No or minimal concerns | Moderate concerns |
|  |  | NA | Study funders reported, but their roles not disclosed. | Researchers' backgrounds reported without reflexivity; similar backgrounds with participants may create power imbalance if researchers were senior. | NA | Unclear on the methodology and theory used. | NA | Study funders reported, but their roles not disclosed. | Researchers' backgrounds reported without reflexivity; similar backgrounds with participants may create power imbalance if researchers were senior. | NA | Researchers' backgrounds reported without reflexivity; similar backgrounds with participants may create power imbalance if researchers were senior; unclear on the methodology and theory used; study funders reported, but their roles not disclosed. |
| 35 | McNamara (2018) | No or minimal concerns | Minor concerns | No or minimal concerns | No or minimal concerns | Minor concerns | Minor concerns | Minor concerns | No or minimal concerns | No or minimal concerns | Minor concerns |
|  |  | NA | Study funders reported, but their roles not disclosed. | NA | NA | No theoretical framework used; no information on managing participants' emotional distress; equity not considered | No reported strategies for preventing or managing participants' emotional distress. | Study funders reported, but their roles not disclosed. | NA | NA | No theoretical framework used; no information on managing participants' emotional distress; equity not considered; study funders reported, but their roles not disclosed |
| 36 | Mkoka (2015) | No or minimal concerns | No or minimal concerns | Serious concerns (unclear) | No or minimal concerns | Minor concerns | No or minimal concerns | No or minimal concerns | Serious concerns (unclear) | No or minimal concerns | Moderate concerns |
|  |  | NA | NA | Researchers' backgrounds and reflexivity not reported. | NA | No specific qualitative methodology reported; no theoretical framework used; no theoretical framework used | NA | NA | Unclear who conducted analysis; researchers' backgrounds and reflexivity not reported. | NA | No specific qualitative methodology reported; no theoretical framework used; no theoretical framework used; unclear who conducted analysis; researchers' backgrounds and reflexivity not reported. |
| 37 | Ndikwetepo (2017) | No or minimal concerns | Minor concerns | No or minimal concerns | No or minimal concerns | Moderate concerns | No or minimal concerns | Minor concerns | No or minimal concerns | No or minimal concerns | Minor concerns |
|  |  | NA | Funder's roles not disclosed | NA | NA | No theoretical framework used; convenience sampling may exclude valuable participants; equity not considered | NA | Funder's roles not disclosed | NA | NA | No theoretical framework used; convenience sampling may exclude valuable participants; equity not considered; funder's roles not disclosed |
| 38 | Neely (2022) | No or minimal concerns | No or minimal concerns | No or minimal concerns | No or minimal concerns | Minor concerns | No or minimal concerns | No or minimal concerns | No or minimal concerns | No or minimal concerns | No or minimal concerns |
|  |  | NA | NA | NA | NA | No theoretical framework used; equity not considered | NA | NA | NA | NA | No theoretical framework used; equity not considered |
| 39 | Oelhafen (2019) | No or minimal concerns | Minor concerns | Serious concerns (unclear) | Minor concerns | Moderate concerns | Minor concerns | Minor concerns | Serious concerns (unclear) | Minor concerns | Moderate concerns |
|  |  | NA | Funder's roles not disclosed | Researchers' backgrounds and reflexivity not reported. | Brief information on context | Data collection process unclear; participant confidentiality measures not reported; qualitative approach appropriate but specific methodology unclear; no theoretical framework used; equity not considered | No reported measures to manage potential emotional risks in participants during interviews on moral distress. | Funder's roles not disclosed | Data collection process and conductors unclear; researchers' backgrounds and reflexivity not reported. | Brief information on context | Data collection process unclear; participant confidentiality measures not reported; qualitative approach appropriate but specific methodology unclear; no theoretical framework used; equity not considered; no reported measures to manage potential emotional risks in participants during interviews on moral distress; funder's roles not disclosed; brief information on context |
| 40 | Rice (2013) | No or minimal concerns | No or minimal concerns | No or minimal concerns | Serious concerns (unclear) | Moderate concerns | No or minimal concerns | No or minimal concerns | No or minimal concerns | Serious concerns (unclear) | Moderate concerns |
|  |  | NA | NA | NA | Study site context not provided | No theoretical framework used; equity/diversity/inclusion not considered; interview locations and attendees unclear | NA | NA | NA | Study site context not provided | No theoretical framework used; equity/diversity/inclusion not considered; interview locations and attendees unclear; Study site context not provided |
| 41 | Rice (2014) | No or minimal concerns | Minor concerns | Serious concerns (unclear) | Serious concerns (unclear) | Moderate concerns | No or minimal concerns | Minor concerns | Serious concerns (unclear) | Serious concerns (unclear) | Moderate concerns |
|  |  | NA | Funder's roles not disclosed | Researchers' backgrounds and reflexivity not reported. | Study site context not provided | No theoretical framework used; no reported strategies for managing participants' emotional risks; recruitment process unclear; equity not considered | NA | Funder's roles not disclosed | Data collection conductors and attendees unclear; researchers' reflexivity and backgrounds not reported. | Study site context not provided | No theoretical framework used; no reported strategies for managing participants' emotional risks; recruitment process unclear; equity not considered; data collection conductors and attendees unclear; researchers' reflexivity and backgrounds not reported; study site context not provided; funder's roles not disclosed |
| 42 | Robertson (2014) | No or minimal concerns | Minor concerns | No or minimal concerns | No or minimal concerns | Minor concerns | No or minimal concerns | Minor concerns | No or minimal concerns | No or minimal concerns | No or minimal concerns |
|  |  | NA | Funder's roles not disclosed | NA | NA | Equity not considered; concerns about data quality collected in participants' homes. | NA | Funder's roles not disclosed | NA | NA | Equity not considered; concerns about data quality collected in participants' homes; funder's roles not disclosed |
| 43 | Sabzevari (2019) | No or minimal concerns | No or minimal concerns | Serious concerns (unclear) | Serious concerns (unclear) | Serious concerns | Moderate concerns | No or minimal concerns | Serious concerns (unclear) | Serious concerns (unclear) | Serious concerns |
|  |  | NA | NA | Lack of reflexivity raises concerns about gender-based power imbalance between male researcher and participants | Context of Iranian setting not provided. | No measures to address potential emotional distress; potential power imbalance between researcher and participants not addressed; research design neglects potential participant emotional distress despite sensitive topic; equity not considered | Inadequate time for participants to consider participation; no measures to address potential emotional distress; potential power imbalance between researcher and participants not addressed | NA | Lack of reflexivity raises concerns about gender-based power imbalance between male researcher and participants | Context of Iranian setting not provided. | No measures to address potential emotional distress; potential power imbalance between researcher and participants not addressed; research design neglects potential participant emotional distress despite sensitive topic; equity not considered; inadequate time for participants to consider participation; context of Iranian setting not provided; lack of reflexivity |
| 44 | Sheen (2022) | No or minimal concerns | No or minimal concerns | Moderate concerns | Minor concerns | Moderate concerns | Moderate concerns | No or minimal concerns | Moderate concerns | Minor concerns | Serious concerns |
|  |  | NA | NA | Impact of researchers' backgrounds on participant relationships and data interpretation not discussed | Lack of information on context | No measures to address potential participant emotional distress; equity not considered; no theoretical framework used | No measure applied to address possible emotional distress in participants | NA | Impact of researchers' backgrounds on participant relationships and data interpretation not discussed | Lack of information on context | Impact of researchers' backgrounds on participant relationships and data interpretation not discussed; no measures to address potential participant emotional distress; equity not considered; no theoretical framework used |
| 45 | Turken (2020) | No or minimal concerns | No or minimal concerns | No or minimal concerns | No or minimal concerns | Moderate concerns | No or minimal concerns | No or minimal concerns | No or minimal concerns | No or minimal concerns | Minor concerns |
|  |  | NA | NA | NA | NA | Concerns about superficial analysis due to brief findings; equity not considered. | NA | NA | NA | NA | Concerns about superficial analysis due to brief findings; equity not considered. |
| 46 | vandenHeuvel (2023) | No or minimal concerns | No or minimal concerns | Minor concerns | No or minimal concerns | Moderate concerns | Moderate concerns | No or minimal concerns | Minor concerns | No or minimal concerns | Moderate concerns |
|  |  | NA | NA | Lack of researchers’ reflexivity | NA | Inclusion/exclusion criteria not reported; no discussion on managing potential participant distress; no theoretical framework used | Concerns about paired interviews potentially influencing responses; no discussion on managing potential participant distress; inclusion/exclusion criteria not reported | NA | Lack of researchers’ reflexivity | NA | Concerns about paired interviews potentially influencing responses; no discussion on managing potential participant distress; inclusion/exclusion criteria not reported; lack of researchers’ reflexivity |
| 47 | Wahlberg (2019) | No or minimal concerns | Minor concerns | Moderate concerns | No or minimal concerns | Moderate concerns | Moderate concerns | Minor concerns | Moderate concerns | No or minimal concerns | Moderate concerns |
|  |  | NA | Funder's roles not disclosed | Researchers' backgrounds and reflexivity not reported. | NA | No strategies reported for managing participant distress; theoretical sampling not used. | No strategies reported for managing participant distress; theoretical sampling not used. | Funder's roles not disclosed | Researchers' backgrounds and reflexivity not reported.; unclear who conducted data analysis | NA | No strategies reported for managing participant distress; theoretical sampling not used; researchers' backgrounds and reflexivity not reported.; unclear who conducted data analysis; funder's roles not disclosed |
| 48 | Wahlberg (2020) | No or minimal concerns | Minor concerns | Moderate concerns | No or minimal concerns | Moderate concerns | Moderate concerns | Minor concerns | Moderate concerns | No or minimal concerns | Minor concerns |
|  |  | NA | Funder's roles not disclosed | Researchers' backgrounds and reflexivity not reported. | NA | No theoretical framework used; no reported strategies for managing potential participant distress. | No reported strategies for managing potential participant distress. | Funder's roles not disclosed | Researchers' backgrounds and reflexivity not reported. | NA | No theoretical framework used; no reported strategies for managing potential participant distress; Researchers' backgrounds and reflexivity not reported; funder's roles not disclosed |
| 49 | Willis (2019) | No or minimal concerns | No or minimal concerns | Serious concerns (unclear) | No or minimal concerns | Serious concerns | Moderate concerns | No or minimal concerns | Serious concerns (unclear) | No or minimal concerns | Serious concerns |
|  |  | NA | NA | Researchers' backgrounds and reflexivity not reported; concerns about possible single-researcher study. | NA | No theoretical framework used; no reported strategies for managing participant distress; possible single-researcher study; concerns that the manager was involved actively in recruitment leading lack of rapport between researchers and participants. | No reported strategies for managing participant distress | NA | Researchers' backgrounds and reflexivity not reported; concerns about possible single-researcher study. | NA | No theoretical framework used; no reported strategies for managing participant distress; possible single-researcher study; concerns that the manager was involved actively in recruitment leading lack of rapport between researchers and participants; researchers' backgrounds and reflexivity not reported |
| 50 | Wright (2018) | No or minimal concerns | No or minimal concerns | Moderate concerns | Serious concerns (unclear) | Minor concerns | Minor concerns | No or minimal concerns | Moderate concerns | Serious concerns (unclear) | Minor concerns |
|  |  | NA | NA | Researchers' backgrounds and reflexivity not reported | No information on context | No theoretical framework used in interpreting data | No theoretical framework used in interpreting data | NA | Researchers' backgrounds and reflexivity not reported | No information on context | Researchers' backgrounds and reflexivity not reported; no theoretical framework used in interpreting data |
| 51 | Young (2015) | No or minimal concerns | No or minimal concerns | Serious concerns (unclear) | No or minimal concerns | Moderate concerns | Moderate concerns | No or minimal concerns | Serious concerns (unclear) | No or minimal concerns | Moderate concerns |
|  |  | NA | NA | Researchers' backgrounds and reflexivity not reported. | NA | No theoretical framework used; no reported strategies for managing participants' emotional distress; unclear who conducted data collection and analysis | No theoretical framework used; no reported strategies for managing participants' emotional distress; unclear who conducted data collection and analysis | NA | Researchers' backgrounds and reflexivity not reported; unclear who conducted data collection and analysis | NA | No theoretical framework used; no reported strategies for managing participants' emotional distress; unclear who conducted data collection and analysis; researchers' backgrounds and reflexivity not reported |
