# Supplementary material for: Well-being of health workers providing maternal and newborn care: A qualitative evidence synthesis
Source: PLOS Glob Public Health. 2026 Feb 11;6(2):e0005522. doi: 10.1371/journal.pgph.0005522 (PMC12893595; doi:10.1371/journal.pgph.0005522)
Supplement: S5 Appendix — (DOCX) [file pgph.0005522.s005.docx]

S5 Appendix. Characteristics of sampled papers

| No | Author (Year) | Title | Publication language | Country | Country Income level | Study Region | Research design | Study setting | Type of health workers |
| --- | --- | --- | --- | --- | --- | --- | --- | --- | --- |
| 1 | Abraham (2020) | A paradox: midwives' experiences of attending a birth resulting in maternal death in a Ghanaian context. | English | Ghana | Lower middle income | Africa | Qualitative | Facility-based | Midwives |
| 2 | Adatara (2021) | Challenges experienced by midwives working in rural communities in the upper east region of Ghana: a qualitative study. | English | Ghana | Lower middle income | Africa | Qualitative | Facility-based | Midwives |
| 3 | Allam (2023) | Midwives' experience of maternal death. | French | Morocco | Lower middle income | Eastern Mediterranean | Qualitative | Facility-based | Midwives |
| 4 | André (2019) | Coping strategies of Norwegian healthcare professionals facing perinatal death: a qualitative study. | English | Norway | High income | Europe | Qualitative | Facility-based | Midwives, Obstetricians, Nurses |
| 5 | Arbour (2020) | The experience of sleep deprivation for midwives practicing in the United States. | English | United States | High income | The Americas | Mixed-methods | Not specified | Midwives |
| 6 | Beck (2012) | A mixed methods study of secondary traumatic stress in labor and delivery nurses. | English | United States | High income | The Americas | Mixed-methods | Not specified | Nurses |
| 7 | Beck (2016) | Vicarious posttraumatic growth in labor and delivery nurses. | English | United States | High income | The Americas | Mixed-methods | Not specified | Nurses |
| 8 | Beck (2020) | Secondary traumatic stress in maternal-newborn nurses: secondary qualitative analysis. | English | United States | High income | The Americas | Qualitative | Not specified | Nurses, Nurse-midwives |
| 9 | Becker (2022) | Silent voices of the midwives: factors that influence midwives' achievement of successful neonatal resuscitation in Sub-Saharan Africa: a narrative inquiry. | English | Tanzania | Lower middle income | Africa | Qualitative | Facility-based | Midwives |
|  | Becker (2023) | Silent tears of midwives: 'I want every mother who gives birth to have her baby alive'-a narrative inquiry of midwives experiences of very early neonatal death from Tanzania. | English | Tanzania | Lower middle income | Africa | Qualitative | Facility-based | Midwives |
| 10 | Bradley (2015) | Too few staff, too many patients: a qualitative study of the impact on obstetric care providers and on quality of care in Malawi. | English | Malawi | Low income | Africa | Qualitative | Facility-based | Midwives, Nurses, Nurse-midwives |
| 11 | Bremnes (2018) | Challenges in day-to-day midwifery practice; a qualitative study from a regional referral hospital in Dar es Salaam, Tanzania. | English | Tanzania | Lower middle income | Africa | Qualitative | Facility-based | Midwives |
| 12 | Calvert (2015) | Trauma and the effects on the midwife | English | New Zealand | High income | Western Pacific | Qualitative | Not specified | Midwives |
| 13 | Cankaya (2021) | Midwives' experiences of witnessing traumatic hospital birth events: a qualitative study. | English | Turkey | Upper middle income | Europe | Qualitative | Facility-based | Midwives |
| 14 | Darling (2020) | Making midwifery services accessible to people of low SES: a qualitative descriptive study of the barriers faced by midwives in Ontario. | English | Canada | High income | The Americas | Qualitative | Facility and community-based | Midwives |
| 15 | Darling (2023) | A mixed-method study exploring barriers and facilitators to midwives' mental health in Ontario. | English | Canada | High income | The Americas | Mixed-methods | Not specified | Midwives |
| 16 | Dartey (2019) | Coping with maternal deaths: the experiences of midwives. | English | Ghana | Lower middle income | Africa | Qualitative | Facility-based | Midwives |
|  | Dartey (2020) | Physical effects of maternal deaths on midwives' health: a qualitative approach. | English | Ghana | Lower middle income | Africa | Qualitative | Facility-based | Midwives |
| 17 | Doherty (2022) | Reducing midwife burnout at organisational level - midwives need time, space and a positive work-place culture. | English | Ireland | High income | Europe | Qualitative | Facility-based | Midwives |
|  | Doherty (2023) | Giving of the self and midwife burnout - an exploration of the consequences of being 'with woman' and how individual midwives can reduce or prevent burnout | English | Ireland | High income | Europe | Qualitative | Facility-based | Midwives |
| 18 | Feeley (2022) | Stories of distress versus fulfilment': a narrative inquiry of midwives' experiences supporting alternative birth choices in the UK national health service. | English | United Kingdom | High income | Europe | Qualitative | Facility and community-based | Midwives |
| 19 | Fontein-Kuipers (2018) | Reports of work-related traumatic events: a mixed methods study. | English | The Netherlands, Belgium | High income | Europe | Mixed-methods | Facility and community-based | Midwives |
| 20 | Geraghty (2019) | Fighting a losing battle: midwives experiences of workplace stress. | English | Australia | High income | Western Pacific | Qualitative | Facility-based | Midwives |
| 21 | Gu (2011) | Chinese midwives' experience of providing continuity of care to labouring women. | English | China | Upper middle income | Western Pacific | Qualitative | Facility-based | Midwives |
| 22 | Hajiesmaello (2022) | Secondary traumatic stress in Iranian midwives: stimuli factors, outcomes and risk management. | English | Iran | Lower middle income | Eastern Mediterranean | Qualitative | Facility-based | Midwives |
| 23 | Halperin (2011) | Stressful childbirth situations: a qualitative study of midwives. | English | Israel | High income | Europe | Qualitative | Facility-based | Midwives |
| 24 | Holly (2019) | Barriers and facilitators of midwives' physical activity behaviour in hospital and community contexts in Scotland. | English | United Kingdom | High income | Europe | Mixed-methods | Facility and community-based | Midwives |
| 25 | Hunter (2014) | Midwives' experiences of workplace resilience. | English | United Kingdom | High income | Europe | Qualitative | Not specified | Midwives |
| 26 | Ismaila (2023) | Midwives' experiences of the consequences of navigating barriers to maternity care. | English | Ghana | Lower middle income | Africa | Qualitative | Facility-based | Midwives |
| 27 | Jaffre (2021) | Being a midwife in West Africa: between sensory experiences, moral standards, socio-technical violence and affective constraints. | English | Benin, Burkina Faso | Lower middle income | Africa | Qualitative | Not specified | Midwives |
| 28 | Kave (2023) | Supporting the needs of midwives caring for women with perinatal loss in South Africa. | English | South Africa | Upper middle income | Africa | Qualitative | Facility-based | Midwives |
| 29 | Lawrence (2024) | "If you need a psychiatrist, it's bad": stigma associated with seeking mental health care among obstetric providers in Ghana. | English | Ghana | Lower middle income | Africa | Qualitative | Facility-based | Midwives, Obstetricians, Nurses |
| 30 | Lawton (2016) | Midwives' experiences of helping women struggling to breastfeed. | English | United Kingdom | High income | Europe | Qualitative | Facility and community-based | Midwives |
| 31 | Long (2013) | Midwives' experiences of work-related shoulder musculoskeletal problems | English | Australia | High income | Western Pacific | Qualitative | Facility-based | Midwives |
| 32 | McNamara (2018) | Intrapartum fetal death and doctors: a qualitative exploration. | English | Ireland | High income | Europe | Qualitative | Facility-based | Obstetricians |
| 33 | Mkoka (2015) | "Once the government employs you, it forgets you": health workers' and managers' perspectives on factors influencing working conditions for provision of maternal health care services in a rural district of Tanzania. | English | Tanzania | Lower middle income | Africa | Qualitative | Facility-based | Nurses |
| 34 | Ndikwetepo (2017) | Midwives' experiences of stress due to emergency childbirths in a Namibian regional hospital. | English | Namibia | Upper middle income | Africa | Qualitative | Facility-based | Midwives |
| 35 | Neely (2022) | Providing maternity care for disadvantaged women in Aotearoa New Zealand: the impact on midwives. | English | New Zealand | High income | Western Pacific | Mixed-methods | Facility and community-based | Midwives |
| 36 | Oelhafen (2019) | Exploring moral problems and moral competences in midwifery: a qualitative study. | English | Switzerland | High income | Europe | Mixed-methods | Facility-based | Midwives, Nurses, Doctors |
| 37 | Rice (2013) | Bearing witness: midwives experiences of witnessing traumatic birth. | English | Australia | High income | Western Pacific | Qualitative | Facility and community-based | Midwives |
| 38 | Rice (2014) | Exploring physical health perceptions, fatigue and stress among health care professionals. | English | Australia | High income | Western Pacific | Mixed-methods | Not specified | Midwives |
| 39 | Robertson (2014) | A phenomenological study of the effects of clinical negligence litigation on midwives in England: the personal perspective. | English | United Kingdom | High income | Europe | Qualitative | Facility-based | Midwives |
| 40 | Sabzevari (2019) | Resilience strategies against working pressures in midwives: a qualitative study. | English | Iran | Lower middle income | Eastern Mediterranean | Qualitative | Facility-based | Midwives |
| 41 | Sheen (2022) | Which events are experienced as traumatic by obstetricians and gynaecologists, and why? a qualitative analysis from a cross-sectional survey and in-depth interviews. | English | United Kingdom | High income | Europe | Mixed-methods | Facility-based | Obstetricians |
| 42 | Turken (2020) | Ethical dilemmas experienced by midwives working in the delivery room | English | Turkey | Upper middle income | Europe | Qualitative | Not specified | Midwives |
| 43 | vandenHeuvel (2023) | Midwives' work-related fear and anxiety and its impact on their wellbeing and performance. a qualitative study of perceived anxiety in community midwives. | English | The Netherlands | High income | Europe | Qualitative | Not specified | Midwives |
| 44 | Wahlberg (2019) | The erratic pathway to regaining a professional self-image after an obstetric work-related trauma: a grounded theory study. | English | Sweden | High income | Europe | Qualitative | Facility-based | Obstetricians |
|  | Wahlberg (2020) | Left alone with the emotional surge - a qualitative study of midwives' and obstetricians' experiences of severe events on the labour ward. | English | Sweden | High income | Europe | Qualitative | Facility-based | Obstetricians |
| 45 | Willis (2019) | Nurses' perspective on caring for women experiencing perinatal loss. | English | United States | High income | The Americas | Qualitative | Facility-based | Nurses |
| 46 | Wright (2018) | Midwifery professional stress and its sources: a mixed-methods study. | English | United States | High income | The Americas | Mixed-methods | Facility and community-based | Midwives |
| 47 | Young (2015) | Burnout: lessons from the lived experience of case loading midwives. | English | New Zealand | High income | Western Pacific | Qualitative | Not specified | Midwives |
