# Supplementary material for: Well-being of health workers providing maternal and newborn care: A qualitative evidence synthesis
Source: PLOS Glob Public Health. 2026 Feb 11;6(2):e0005522. doi: 10.1371/journal.pgph.0005522 (PMC12893595; doi:10.1371/journal.pgph.0005522)
Supplement: S6 Appendix — (DOCX) [file pgph.0005522.s006.docx]

# S6 Appendix. Characteristics of papers awaiting classification

| No | Author (Year) | Title | Country | Country Income level | Study region | Research design | Study setting | Type of health workers |
| --- | --- | --- | --- | --- | --- | --- | --- | --- |
| 1 | Al-Otaibi (2024) | Perceived structural empowerment, resilience, and intent to stay among midwives and registered nurses in Saudi Arabia: a convergent parallel mixed methods study. | Saudi Arabia | High income | Eastern Mediterranean | Mixed-methods | Facility-based | Midwives, Nurses |
| 2 | Alsaraireh (2025) | A qualitative study of contributing factors to burnout among Jordanian midwives. | Jordan | Lower middle income | Eastern Mediterranean | Qualitative | Facility-based | Midwives |
| 3 | Anchors (2024) | A mixed-methods stress audit with midwives in the United Kingdom. | United Kingdom | High income | Europepe | Mixed-methods | Community-based | Midwives |
| 4 | Ani-Amponsah (2024) | Midwives' perspectives on rural birthing experiences and newborn survival in Ghana. | Ghana | Lower middle income | Africa | Qualitative | Community-based | Midwives |
| 5 | Aseffa (2024) | Disrupting racism in Ontario midwifery | Canada | High income | The Americas | Qualitative | Community-based | Midwives |
| 6 | Bayrı Bing¨ol (2024) | Partners in pain, two sides of a zipper - midwives' experiences with stillbirth: a qualitative study. | Türkiye | Upper middle income | Europepe | Qualitative | Facility-based | Midwives |
| 7 | Boulton (2024) | "Burnout is real": a SWOT analysis of Albertan midwives' perspectives on providing midwifery care, | Canada | High income | The Americas | Qualitative | Community-based | Midwives |
| 8 | Buchanan (2025) | The work of midwives: the socio-institutional theory of the meaning of midwives' work-life balance. | Australia | High income | Western Pacific | Qualitative | Community-based | Midwives |
| 9 | Carvajal (2024) | Chilean midwives' experiences while providing induced abortion care after the enactment of a law decriminalising abortion. | Chile | High income | The Americas | Qualitative | Community-based | Midwives |
| 10 | Cheung (2024) | Healthcare practitioners' experiences and perspectives of music in perinatal care in Ireland: an exploratory survey. | Ireland | High income | Europepe | Mixed-methods | Community-based | Midwives, Obstetrician- gynaecologist |
| 11 | Comert (2025) | Midwives' negative experiences and outcomes, a phenomenological study "Being a midwife in Istanbul; I am a midwife, I accompany births…, but I cannot be a midwife!" | Türkiye | Upper middle income | Europepe | Qualitative | Community-based | Midwives |
| 12 | Cummins (2025) | The continuity relationship makes caring for women with anxiety and depression easier, but it is also a heavy responsibility. | Australia | High income | Western Pacific | Mixed-methods | Facility-based | Midwives |
| 13 | Daellenbach (2024) | Midwifery mentorship in Aotearoa New Zealand: the mentors’ perspective. | New Zealand | High income | Western Pacific | Qualitative | Community-based | Midwives |
| 14 | Dartey (2025) | The experiences of midwives in the management of obstetric emergencies: a phenomenological study in the Kwahu West Municipality, Ghana. | Ghana | Lower middle income | Africa | Qualitative | Facility-based | Midwives |
| 15 | Dartey (2024) | Experiences of nurses and midwives with indecorously structured duty rosters at selected health facilities in Ho, Volta Region of Ghana: a qualitative study. | Ghana | Lower middle income | Africa | Qualitative | Community-based | Midwives, Nurses |
| 16 | Denton (2024) | Supporting older nurses and midwives in the Australian healthcare workplace-a qualitative descriptive study. | Australia | High income | Western Pacific | Qualitative | Community-based | Midwives, Nurses |
| 17 | Donnelly (2024) | Understanding attrition of early career midwives in Australia. | Australia | High income | Western Pacific | Qualitative | Community-based | Midwives |
| 18 | Exell (2025) | Factors positively influencing GP obstetricians to remain in rural and remote obstetric practice. | Australia | High income | Western Pacific | Qualitative | Community-based | GP Obstetricians |
| 19 | Garti (2024) | Midwives' experiences of providing pre-eclampsia care in a low- and middle-income country - a qualitative study. | Ghana | Lower middle income | Africa | Qualitative | Facility-based | Midwives |
| 20 | Gauci (2025) | The lived experiences of newly qualified midwives in supporting women during labor and birth: a hermeneutic phenomenological study in Malta | Malta | High income | Europepe | Qualitative | Facility-based | Midwives |
| 21 | Halperin (2024) | Predicting post-traumatic stress disorder: the complex relationship between burnout, intentions to leave and emotional support among health care professionals. | Israel | High income | Europepe | Mixed-methods | Facility-based | Nurses-midwives, Gynaecologists |
| 22 | Hewitt (2024) | The sustainability of midwifery group practice: a cross-sectional study of midwives and managers. | Australia | High income | Western Pacific | Mixed-methods | Community-based | Midwives |
| 23 | Holton (2024) | Developing nurse and midwife centred rostering principles using co-design: a mixed-methods study. | Australia | High income | Western Pacific | Mixed-methods | Facility-based | Midwives, Nurses |
| 24 | Ismaila (2024) | Midwives' experiences of the consequences of navigating barriers to maternity care. | Ghana | Lower middle income | Africa | Qualitative | Facility-based | Midwives |
| 25 | Kakyo (2019) | Exploring the dark side of informal mentoring: experiences of nurses and midwives working in hospital settings in Uganda. | Uganda | Low income | Africa | Qualitative | Facility-based | Midwives, Nurses |
| 26 | Karaçay yikar (2024) | Women's health nurses' compassion fatigue, difficulties experienced and coping experiences regarding exit methods: a qualitative study | Türkiye | Upper middle income | Europepe | Qualitative | Facility-based | Nurses |
| 27 | Lips (2024) | Bridging perspectives, building resilience: safety-II guided reflexive dialogues between care professionals and clients as part of developing integrated maternity care. | The Netherlands | High income | Europepe | Qualitative | Community-based | Midwives, Nurses, GPs, Obstetricians, Paediatricians |
| 28 | Liu (2024) | Experience among postnatal nurses with two or three children returning to work within 3 months in China: a qualitative study. | China | Upper middle income | Western Pacific | Qualitative | Facility-based | Nurses |
| 29 | MacLeod (2025) | Orchestrating care for a good life event: a hermeneutic study of the overlooked practices of rural perinatal nurses. | Canada | High income | The Americas | Qualitative | Facility-based | Nurses |
| 30 | McElroy (2024) | The experiences and perceptions of rural and remote nurses who provide care to pregnant women in the absence of midwives. | Australia | High income | Western Pacific | Qualitative | Community-based | Nurses |
| 31 | Miao (2024) | To explore the impact of traumatic birth experiences on midwives' experience of empathy: a qualitative study. | China | Upper middle income | Western Pacific | Qualitative | Facility-based | Midwives |
| 32 | Milku (2024) | Challenges and coping strategies for providing maternal health care services among health care professionals in rural health facilities in Wolaita Zone, Southern Ethiopia: a qualitative study. | Ethiopia | Lower middle income | Africa | Qualitative | Community-based | Midwives, Nurses, GPs |
| 33 | Pérez-Castejón (2025) | Job satisfaction among midwives in high-intervention birthing rooms: a qualitative phenomenological study. | Spain, Argentina | High income, Upper middle income | Europe, The Americas | Qualitative | Community-based | Midwives |
| 34 | Pezaro (2025) | Exploring midwives' and nurse-midwives' professional identity and how midwifery may be best represented in the public realm: a global convergent parallel mixed-methods study | Global | NA | NA | Mixed-methods | Community-based | Midwives, Nurse- midwives |
| 35 | Steen (2025) | Compassionate self-care for nurses and midwives: a sequential explanatory mixed methods study. | Australia | High income | Western Pacific | Mixed-methods | Community-based | Midwives, Nurses |
| 36 | Temane (2024) | Midwives' lived experiences of caring for women with mobility disabilities during pregnancy, labour and puerperium in Eswatini: a qualitative study | Eswatini | Lower middle income | Africa | Qualitative | Facility-based | Midwives |
| 37 | Temesgen (2025) | Challenges, roles, and capacity of midwives in providing maternal health services in public health facilities in Addis Ababa, Ethiopia: a qualitative analysis. | Ethiopia | Lower middle income | Africa | Qualitative | Facility-based | Midwives |
| 38 | Terry (2025) | Early career midwives' experiences of development opportunities and their relation to retention and job satisfaction: an interpretative phenomenological analysis study. | United Kingdom | High income | Europepe | Qualitative | Community-based | Midwives |
| 39 | Thumm (2024) | The relationship between restrictive regulation of midwives, practice environment, and professional burnout: a 7-state mixed-methods comparison of autonomous and restrictive state regulation. | United States | High income | The Americas | Mixed-methods | Community-based | Midwives, Nurse-midwives |
| 40 | Thumm (2025) | The decision to leave the midwifery workforce in the United States: a qualitative investigation | United States | High income | The Americas | Qualitative | Community-based | Midwives, Nurse-midwives |
| 41 | Tseer (2024) | Experiences of workplace conflicts by midwives and implications for their wellbeing in selected hospitals in the Northern Region of Ghana. | Ghana | Lower middle income | Africa | Qualitative | Community-based | Midwives |
| 42 | Wood (2025) | Enhancing maternity healthcare workers’ wellbeing using insider participatory action research | United Kingdom | High income | Europepe | Qualitative | Community-based | Midwives, Obstetric doctors, Anaesthetic doctors |
| 43 | Yang (2025) | Emotional labor and coping strategies of gynecological nurses in recurrent pregnancy loss care: a qualitative phenomenological study. | China | Upper middle income | Western Pacific | Qualitative | Community-based | Nurses |
