# Supplementary material for: Well-being of health workers providing maternal and newborn care: A qualitative evidence synthesis
Source: PLOS Glob Public Health. 2026 Feb 11;6(2):e0005522. doi: 10.1371/journal.pgph.0005522 (PMC12893595; doi:10.1371/journal.pgph.0005522)
Supplement: S7 Appendix — (DOCX) [file pgph.0005522.s007.docx]

S7 Appendix. GRADE-CERQual Evidence Profile

| **Findings** | **Summary of qualitative findings** | **Contributing qualitative studies** | **Methodological limitations** | **Coherence** | **Relevance** | **Adequacy** | **Overall CERQual assessment** | **Explanation of overall assessment** |  |  |  |  |  |  |  |
| --- | --- | --- | --- | --- | --- | --- | --- | --- | --- | --- | --- | --- | --- | --- | --- |
| Overarching Theme 1: Factors influencing health workers’ well-being | | | | | | | | |  |  |  |  |  |  |  |
| Theme 1: Meaningful relationships have impact | | | | | | | | |  |  |  |  |  |  |  |
| 1 | **Valuing the relationship between health workers and women and their families.** Health workers valued the trust and relationships they built with women and their families. These relationships led to both positive emotional experiences and, at times, significant emotional strain. | (34, 36, 38, 42, 43, 45, 49-53, 56-58, 60, 62, 64, 67, 69, 70, 75-77, 80, 81, 83) | Moderate concerns:  2 papers with no or minor concerns, 7 with minor concerns (fit between research design and researchers, fit between research conduct and researchers, fit between research design and context), 9 with moderate concerns (fit between research design and researchers, fit between research conduct and researchers, fit between research design and context), 8 with serious concerns (fit between research design and researchers, fit between research conduct and researchers, fit between research design and research conduct, fit between research conduct and research aim/questions, fit between research conduct and context, fit between research design domains and research aim/ questions) | No or very minor concerns | Minor concerns:  13 out of 26 papers are indirectly relevant to the review aim. Contributing papers represented 18 countries in 5 regions, AFRO (Ghana, Tanzania, Benin, Burkina Faso, South Africa, Namibia), EMRO (Morocco, Iran), PAHO (USA, Canada), WPRO (New Zealand, Australia, China ), EURO (Ireland, UK, Switzerland, The Netherlands, Sweden), where 9 are high income, 3 upper middle income, and 6 lower middle income. All perspectives came from health workers (midwives, nurses, nurse-midwives, obstetricians, doctors). | Minor concerns: 26 out of 51 papers contributed to the review finding (15 thick and 11 thin data). | Moderate confidence | No or very minor concerns on coherence, minor concerns on relevance (13 papers with indirectly relevant aim),  minor concerns on adequacy (26 of 51 papers contributed; 15 moderately thick and 11 thin data), moderate concerns on methodological limitations (fit between research design and researchers, fit between research conduct and researchers, fit between research design and research conduct, fit between research conduct and research aim/questions, fit between research conduct and context, fit between research design domains and research aim/ questions) |  |  |  |  |  |  |  |
| 2 | **Experiencing the emotional toll of adverse events.** Maternal and neonatal complications or deaths were traumatic events for health workers, with significant negative and long-lasting impacts on their mental and physical health. Health workers often questioned their care decisions, felt a heavy sense of responsibility for adverse outcomes, experienced stressful flashbacks, and had difficulty coping with their emotions. | (34, 36-38, 40, 43, 44, 46, 47, 49, 51, 53-58, 62, 64-66, 70, 72, 75-78, 81-83) | Moderate concerns:  1 paper with no or minor concerns, 9 with minor concerns (Fit between Research design domains and Research conduct domain, Fit between Research design domains and Researchers, Fit between Research conduct domains and Researchers, Fit between Research design domains and Context, Fit between Research conduct domains and Context ), 9 with moderate concerns (Fit between Research design domains and Researchers, Fit between Research conduct domains and Researchers, Fit between Research design domains and Context, Fit between Research conduct domains and Context), 11 with serious concerns (Fit between Research design domains and Researchers, Fit between Research conduct domains and Researchers, Fit between Research design domains and Context, Fit between Research conduct domains and Context, Fit between Research design domains and Research conduct domain, Fit between Research conduct domains and Research aim/questions, Fit between Research conduct domains and Stakeholders) | No or very minor concerns | Minor concerns:  12 out of 30 papers are indirectly relevant to the review aim. Contributing papers represented 21 countries in 5 regions, EURO (Norway, Turkey, Ireland, UK, The Netherlands, Belgium, Switzerland, Sweden, Israel), AFRO (Ghana, Tanzania, Benin, Burkina Faso, South Africa, Namibia ), PAHO (USA, Canada), WPRO (New Zealand, Australia), EMRO (Morocco, Iran), where 13 are high income, 4 upper middle income, and 6 lower middle income. All perspectives came from health workers (midwives, nurses, nurse-midwives, obstetricians, doctors). | No or very minor concerns | Moderate confidence | No or very minor concerns on coherence, no or very minor concerns on adequacy, minor concerns on relevance (12 papers with indirectly relevant aim), moderate concerns on methodological limitations (fit between research design domains and researchers, fit between research conduct domains and researchers, fit between research design domains and context, fit between research conduct domains and context, fit between research design domains and research conduct domain, fit between research conduct domains and research aim/questions, fit between research conduct domains and stakeholders) |  |  |  |  |  |  |  |
| **Theme 2: The impact of working conditions** | | | | | | | | |  |  |  |  |  |  |  |
| 3 | **Needing fair and equitable remuneration.** Fair and equitable remuneration across different professions and service areas (e.g., rural or urban) was crucial for midwives and nurses to feel valued and respected. When pay was unfair and inequitable, they felt unrecognised, undervalued, frustrated and demotivated. | (35, 42, 57, 63, 77) | Minor concerns:  3 with minor concerns (Fit between Research design domains and Researchers, Fit between Research conduct domains and Researchers), 2 with moderate concerns (Fit between Research design domains and Researchers, Fit between Research conduct domains and Researchers, Fit between Research design domains and Research conduct domain) | No or very minor concerns | Moderate concerns:  4 out of 5 papers are indirectly relevant to the review aim. Contributing papers represented 5 countries in 2 regions, AFRO (Ghana, Tanzania, Benin, Burkina Faso), PAHO (Canada), where 4 are lower middle income and 1 is high income. Perspectives came from midwives and nurses. | Serious concerns: 5 out of 51 papers contributed to the review finding (3 moderately thick and 2 thin data). | Low confidence | No or very minor concerns on coherence, minor concerns on methodological limitations (fit between research design domains and researchers, fit between research conduct domains and researchers, fit between research design domains and research conduct domain), moderate concerns on relevance (4 papers with indirectly relevant aim, all papers only represented two regions), serious concerns on adequacy (5 out of 51 papers contributed; 3 moderately thick and 2 thin data to review finding, 3 with moderate data) |  |  |  |  |  |  |  |
| 4 | **Struggling with unhealthy workplace culture.** Some health workers perceived that an unhealthy workplace culture contributed to their experiences of mental distress, powerlessness, isolation, burnout and poor job performance and satisfaction. | (37, 38, 40, 41, 43-45, 48, 50, 51, 54-68, 70-74, 77, 81, 83, 84) | Moderate concerns:  2 papers with no or minor concerns, 14 with minor concerns (Fit between Research design domains and Researchers, Fit between Research design domains and Context, Fit between Research conduct domains and Researchers, Fit between Research conduct domains and Context), 9 with moderate concerns (Fit between Research design domains and Researchers, Fit between Research design domains and Context, Fit between Research conduct domains and Researchers, Fit between Research conduct domains and Context ), 9 with serious concerns (Fit between Research design domains and Researchers, Fit between Research design domains and Context, Fit between Research conduct domains and Researchers, Fit between Research conduct domains and Context, Fit between Research design domains and Stakeholders, Fit between Research conduct domains and Stakeholders, Fit between Research design domains and Research conduct domain, Fit between Research conduct domains and Research aim/questions) | No or very minor concerns | Minor concerns:  17 out of 34 papers are indirectly relevant to the review aim. Contributing papers represented 19 countries in 5 regions, EURO (Norway, Ireland, UK, Israel, Switzerland, Sweden, Turkey), PAHO (USA, Canada), WPRO (New Zealand, Australia), EMRO (Iran), AFRO (Tanzania, Malawi, Ghana, Benin, Burkina Faso, South Africa, Namibia), where 11 are high income, 3 upper middle income, 5 lower middle income, and 1 low income. Perspectives came from midwives and nurses. All perspectives came from health workers (midwives, nurses, nurse-midwives, obstetricians, doctors). | No or very minor concerns | Moderate confidence | No or very minor concerns on coherence, no or very minor concerns on adequacy, minor concerns on relevance (17 papers with indirectly relevant aim), moderate concerns on methodological limitations (fit between research design domains and researchers, fit between research design domains and context, fit between research conduct domains and researchers, fit between research conduct domains and context, fit between research design domains and stakeholders, fit between research conduct domains and stakeholders, fit between research design domains and research conduct domain, fit between research conduct domains and research aim/questions) |  |  |  |  |  |  |  |
| 5 | **Dealing with heavy workloads.** Heavy workloads, exacerbated by staff shortages, inadequate skills, and poor workload management, resulted in emotional distress, burnout, and physical injuries. These negative emotional experiences were further intensified by the inability to take leave or by feelings of guilt associated with taking time off, as it could place additional burdens on their colleagues. | (35, 41, 42, 44, 45, 48, 49, 51, 52, 55, 56, 58, 61, 63, 64, 71, 73, 74, 77, 81, 82) | Moderate concerns:  1 paper with no or minor concerns, 7 with minor concerns (Fit between Research design domains and Researchers, Fit between Research conduct domains and Researchers, Fit between Research design domains and Research conduct domain, Fit between Research conduct domains and Research aim/questions), 9 with moderate concerns (Fit between Research design domains and Researchers, Fit between Research design domains and Context, Fit between Research conduct domains and Researchers, Fit between Research conduct domains and Context, Fit between Research design domains and Research conduct domain), 4 with serious concerns (Fit between Research design domains and Researchers, Fit between Research conduct domains and Researchers, Fit between Research design domains and Research conduct domain, Fit between Research conduct domains and Stakeholders, Fit between Research conduct domains and Research aim/questions) | No or very minor concerns | Minor concerns:  10 out of 21 papers are indirectly relevant to the review's aim. Contributing papers represented 15 countries in 4 regions, AFRO (Ghana, Malawi, Tanzania, South Africa, Namibia), EURO (Ireland, UK, Switzerland, Sweden, Turkey), WPRO (Australia, China, New Zealand), PAHO (USA, Canada), where 8 are high income, 4 are upper middle income, 2 are lower middle income and 1 low income. All perspectives came from health workers (midwives, nurses, nurse-midwives, obstetricians, doctors). | No or very minor concerns | Moderate confidence | No or very minor concerns on coherence, no or very minor concerns on adequacy, minor concerns on relevance (10 papers with indirectly relevant aim), moderate concerns on methodological limitations (fit between research design domains and researchers, fit between research conduct domains and researchers, fit between research design domains and research conduct domain, fit between research conduct domains and stakeholders, fit between research conduct domains and research aim/questions) |  |  |  |  |  |  |  |
| 6 | **Finding opportunities for professional growth.** Opportunities for growth, including training, further education, and career advancement, were positive motivators for midwives. However, their access to these opportunities was limited when health facilities faced staff shortages or implemented unpredictable scheduling systems. | (35, 42, 52) | Minor concerns:  1 paper with minor concerns (Fit between Research design domains and Researchers, Fit between Research conduct domains and Researchers), 2 with moderate concerns (Fit between Research design domains and Researchers, Fit between Research conduct domains and Researchers, Fit between Research design domains and Research conduct domain) | No or very minor concerns | Serious concerns:  All three papers are indirectly relevant to the review's aim. Contributing papers represented 3 countries in 2 regions, AFRO (Ghana, Tanzania), WPRO (China), where 2 are lower middle income and 1 is upper middle income. All perspectives came from midwives. | Serious concerns: 3 out of 51 papers contributed to the review finding (2 moderate and 1 thin data). | Very low confidence | No or very minor concerns on coherence, minor concerns on methodological limitations (fit between research design domains and researchers, fit between research conduct domains and researchers, fit between research design domains and research conduct domain), serious concerns on relevance (all papers with indirectly relevant aim, perspectives came only from midwives in LMICs), serious concerns on adequacy (3 out of 51 papers contributed; 2 moderately thick and 1 thin to the review finding with moderate to thin data) |  |  |  |  |  |  |  |
| 7 | **Working with inadequate infrastructure.** Insufficient infrastructure in health facilities, including the absence of clean running water and shortages of essential equipment and supplies, heightened the risk of infections and physical injuries among midwives and nurses, leading to frustration as it hindered their ability to deliver high-quality care to women and their families. Inadequate infrastructure also negatively impacted midwives and nurses’ physical and psychological health. | (35, 40, 42, 56-58, 61, 63, 64, 74, 79, 82). | Moderate concerns:  4 papers with minor concerns (Fit between Research design domains and Researchers, Fit between Research conduct domains and Researchers, Fit between Research design domains and Research conduct domain ), 5 with moderate concerns (Fit between Research design domains and Researchers, Fit between Research design domains and Context, Fit between Research conduct domains and Researchers, Fit between Research conduct domains and Context), 3 with serious concerns (Fit between Research design domains and Research conduct domain, Fit between Research conduct domains and Research aim/questions, Fit between Research design domains and Researchers, Fit between Research conduct domains and Researchers ) | No or very minor concerns | Minor concerns:  7 out of 12 papers are indirectly relevant to the review aim. Contributing papers represented 9 countries in 4 regions: AFRO (Ghana, Tanzania, Benin, Burkina Faso, South Africa, Namibia), PAHO (USA), EURO (UK), and WPRO (Australia), where 3 are high income, 2 are upper middle income, and 4 are lower middle income. All perspectives came from midwives. | Moderate concerns:  12 out of 51 papers contributed to the review finding (6 moderate and 6 thin data). | Moderate confidence | No or very minor concerns on coherence, minor concerns on relevance (7 papers with indirectly relevan aim, represented midwives perspectives only), moderate concerns on adequacy (12 of 51 papers contributed; 6 moderate and 6 thin data), moderate concerns on methodological limitations (fit between research design domains and research conduct domain, fit between research conduct domains and research aim/questions, fit between research design domains and researchers, fit between research conduct domains and researchers). |  |  |  |  |  |  |  |
| **Theme 3: Societal and legal pressures** | | | | | | | | |  |  |  |  |  |  |  |
| 8 | **Facing socio-gendered challenges in rural areas.** Rural postings provided midwives and nurses with opportunities for quicker career advancement, but they also presented challenges, including poor living conditions, inadequate housing, limited access to quality education, and safety risks. For female health workers, these risks were even more pronounced due to sexist and gendered power dynamics, which could adversely impact their career progression and transfer requests. | (35, 56, 57, 63, 80) | Moderate concerns:  1 paper with no or minor concerns, 2 with minor concerns (Fit between Research design domains and Researchers, Fit between Research conduct domains and Researchers), 1 with moderate concerns (Fit between Research design domains and Researchers, Fit between Research conduct domains and Researchers ), 1 with serious concerns (Fit between Research design domains and Research conduct domain, Fit between Research design domains and Research aim/ questions, Fit between Research conduct domains and Research aim/ questions)s | No or very minor concerns | Moderate concerns:  All papers are indirectly relevant to the review aim. Contributing papers represented 5 countries in 2 regions, AFRO (Ghana, Benin, Burkina Faso, Tanzania), WPRO (New Zealand), where 1 is high income and 4 are lower middle income. Perspectives came from midwives and nurses. | Serious concerns: 5 out of 51 papers contributed to the review finding (4 moderate and 1 thin data). | Low confidence | No or very minor concerns on coherence, moderate concerns on relevance (all papers with indirectly relevant aim, represented two regions), moderate concerns on methodological limitations (fit between research design domains and research conduct domain, fit between research design domains and research aim/ questions, fit between research conduct domains and research aim/ questions, fit between research design domains and researchers, fit between research conduct domains and researchers), serious concerns on adequacy (5 of 51 papers contributed; 4 moderately thick and 1 thin data) |  |  |  |  |  |  |  |
| 9 | **Encountering negative or inaccurate media portrayals.** Media coverage blaming midwives and obstetricians for maternal or newborn injury or death made health workers feel upset, frustrated and powerless. Health workers felt these reports were often inaccurate, but they were unable to share their perspectives due to professional confidentiality and limited support from their employers. | (62, 64, 70, 71) | Minor concerns:  3 papers with minor concerns (Fit between Research design domains and Research conduct domain, Fit between Research conduct domains and Research aim/questions), 1 with moderate concerns (Fit between Research design domains and Researchers, Fit between Research design domains and Research conduct domain, Fit between Research conduct domains and Research aim/questions, Fit between Research conduct domains and Researchers) | No or very minor concerns | Moderate concerns:  3 out of 4 papers are indirectly relevant to the review aim. Contributing papers represented 3 countries in 2 regions, AFRO (Namibia), EURO (Ireland, Sweden), where 2 are high income and 1 is upper middle income. Perspectives came from midwives and obstetricians. | Serious concerns: 4 out of 51 papers contributed to the review finding (2 thick and 2 thin data). | Low confidence | No or very minor concerns on coherence, minor concerns on methodological limitations (fit between research design domains and researchers, fit between research design domains and research conduct domain, fit between research conduct domains and research aim/questions, fit between research conduct domains and researchers), moderate concerns on relevance (3 papers with indirectly relevant aim, represented two regions), serious concerns on adequacy (4 of 51 papers contributed; 2 thick and 2 thin data) |  |  |  |  |  |  |  |
| 10 | **Fearing mental health stigma.** Mental health stigma related to both sharing experiences and accessing support meant some health workers were reluctant to seek help for themselves. Concerns about a lack of confidentiality when seeking mental health care further contributed to this hesitation, leading to many health workers choosing to consult with senior colleagues rather than pursue professional support**.** | (59, 69) | Moderate concerns:  1 paper with minor concerns (Fit between Research design domains and Research conduct domains, Fit between Research conduct domains and Context), 1 with moderate concerns (Fit between Research design domains and Research conduct domain, Fit between Research conduct domains and Research aim/questions) | No or very minor concerns | Moderate concerns:  All papers are directly relevant to the review aim. Contributing papers represented 2 countries in 2 regions, AFRO (Ghana), EURO (The Netherlands), where 1 is high income and 1 is lower middle income. Perspectives came from midwives, obstetricians and nurses. | Serious concerns: 2 out of 51 papers contributed to the review finding with thick data | Low confidence | No or very minor concerns on coherence, moderate concerns on relevance (all papers represented 2 countries), moderate concerns on methodological limitations (fit between research design domains and research conduct domain, fit between research conduct domains and context, fit between research conduct domains and research aim/questions), serious concerns on adequacy (2 of 51 papers contributed with moderately thick data ) |  |  |  |  |  |  |  |
| 11 | **Experiencing pressure from litigation and investigation.** Clinical audits, lawsuits, and professional investigations affected health workers' mental and physical health, with the impact worsened by perceived unfair processes. During these proceedings, health workers reported that they did not receive adequate legal and emotional support from their employers. | (34, 36, 38, 44, 56, 64, 66, 70, 71, 83, 84) | Moderate concerns:  1 paper with no or minor concerns, 3 with minor concerns (Fit between Research design domains and Context, Fit between Research conduct domains and Context ), 3 with moderate concerns (Fit between Research design domains and Researchers, Fit between Research conduct domains and Researchers, Fit between Research design domains and Context, Fit between Research conduct domains and Context ), 4 with serious concerns (Fit between Research design domains and Researchers, Fit between Research conduct domains and Researchers, Fit between Research design domains and Context, Fit between Research conduct domains and Context, Fit between Research design domains and Research conduct domain, Fit between Research conduct domains and Research aim/questions, Fit between Research design domains and Research aim/questions) | No or very minor concerns | Moderate concerns:  11 out of 51 papers contributed to the review finding (6 moderately thick and 5 thin data) | Moderate concerns:  11 out of 51 papers contributed to the review finding (6 moderately thick and 5 thin data) | Moderate confidence | No or very minor concerns on coherence, minor concerns on relevance (4 papers with indirectly relevant aim), moderate concerns on adequacy (11 of 51 papers contributed; 6 moderately thick and 5 thin data), moderate concerns on methodological limitations (fit between research design domains and researchers, fit between research conduct domains and researchers, fit between research design domains and context, fit between research conduct domains and context, fit between research design domains and research conduct domain, fit between research conduct domains and research aim/questions, fit between research design domains and research aim/questions) |  |  |  |  |  |  |  |
| **Overarching Theme 2: Foundations of well-being and resilience in health workers** | | | | | | | | |  |  |  |  |  |  |  |
| **Theme 4: Personal resilience** | | | | | | | | |  |  |  |  |  |  |  |
| 12 | **Finding strength in professional identities.** Professional identities were crucial in promoting resilience. Professional identities were shaped by cognitive, emotional, and relational dimensions, involving autonomy, belonging, fulfilment, achievement, responsibility, passion, compassion for patients, confidence in their abilities, and self-efficacy. | (40, 43, 45, 49-57, 60, 62, 64-69, 72, 73, 77, 80-82) | Moderate concerns:  3 papers with no or minor concerns, 10 with minor concerns (Fit between Research design domains and Researchers, Fit between Research conduct domains and Researchers, Fit between Research design domains and Context, Fit between Research conduct domains and Context), 7 with moderate concerns (Fit between Research design domains and Researchers, Fit between Research design domains and Context, Fit between Research conduct domains and Researchers, Fit between Research conduct domains and Context), 6 with serious concerns (Fit between Research design domains and Stakeholders, Fit between Research design domains and Researchers, Fit between Research design domains and Research conduct domain, Fit between Research conduct domains and Stakeholders, Fit between Research conduct domains and Researchers, Fit between Research conduct domains and Context, Fit between Research design domains and Research aim/questions, Fit between Research conduct domains and Research aim/questions, Fit between Research design domains and Context) | No or very minor concerns | Minor concerns:  13 out of 26 papers are indirectly relevant to the review aim. Contributing papers represented 17 countries in 5 regions, AFRO (Tanzania, Ghana, Benin, Burkina Faso, Namibia, EMRO(Iran), EURO (Ireland, UK, Israel, Switzerland, Turkey, The Netherlands), PAHO(Canada, USA), WPRO (New Zealand, Australia, China), where 9 are high income, 3 are upper middle income and 5 are lower middle income. Perspectives came from midwives, obstetricians, nurses, and doctors. | No or very minor concerns:  26 out of 51 papers contributed to the review finding (22 moderately thick and 4 thin data) | Moderate confidence | No or very minor concerns on coherence, no or very minor concerns on adequacy, minor concerns on relevance (13 out of 26 papers with indirectly relevant aim), moderate concerns on methodological limitations (fit between research design domains and stakeholders, fit between research design domains and researchers, fit between research design domains and research conduct domain, fit between research conduct domains and stakeholders, fit between research conduct domains and researchers, fit between research conduct domains and context, fit between research design domains and research aim/questions, fit between research conduct domains and research aim/questions, fit between research design domains and context) |  |  |  |  |  |  |  |
| 13 | **Valuing spirituality.** Spiritual beliefs and practices enabled some health workers to cope with significant challenges, such as maternal and neonatal deaths, which fostered resilience and provided a sense of purpose. | (34, 36, 39, 58, 67, 72, 75, 76) | Moderate concerns:  1 paper with no or minor concerns, 3 with moderate concerns (Fit between Research design domains and Researchers, Fit between Research conduct domains and Researchers, Fit between Research design domains and Context, Fit between Research conduct domains and Context ), 4 with serious concerns (Fit between Research design domains and Researchers, Fit between Research conduct domains and Researchers, Fit between Research design domains and Research conduct domain, Fit between Research conduct domains and Research aim/questions, Fit between Research design domains and Context, Fit between Research conduct domains and Context) | No or very minor concerns | Minor concerns:  3 out of 8 papers are indirectly relevant to the review aim. Contributing papers represented 6 countries in 3 regions, AFRO (Ghana, Tanzania, South Africa), EMRO (Iran, Morocco), and PAHO (USA), where 1 is high income, 1 is upper middle income, and 4 are lower middle income. Perspectives came from midwives and nurses. | Moderate concerns:  8 out of 51 papers contributed to the review finding (5 moderate and 3 thin data) | Moderate confidence | No or very minor concerns on coherence, minor concerns on relevance (3 papers with indirectly relevant aim), moderate concerns on adequacy (8 of 51 papers contributed; 5 moderate and 3 thin data), moderate concerns on methodological limitations (fit between research design domains and researchers, fit between research conduct domains and researchers, fit between research design domains and research conduct domain, fit between research conduct domains and research aim/questions, fit between research design domains and context, fit between research conduct domains and context), |  |  |  |  |  |  |  |
| 14 | **Achieving work-life harmony.** Achieving work-life harmony was seen as a potential solution to reduce burnout and alleviate mental and physical distress among health workers, ultimately helping to build their resilience. However, several factors hindered this, including unclear boundaries between personal and professional lives, expectations to be constantly available, physical injuries, personal caregiving responsibilities, inadequate workload management, exposure to adverse events, and long commutes to work. | (36, 38, 41, 42, 44, 46, 47, 49, 51-57, 61, 72-74, 77-82, 84) | Moderate concerns:  1 paper with no or minor concerns, 7 with minor concerns (Fit between Research design domains and Researchers, Fit between Research conduct domains and Researchers, Fit between Research design domains and Context, Fit between Research conduct domains and Context), 8 with moderate concerns (Fit between Research design domains and Researchers, Fit between Research design domains and Context, Fit between Research conduct domains and Researchers, Fit between Research conduct domains and Context, Fit between Research design domains and Research conduct domain), 10 with serious concerns (Fit between Research design domains and Researchers, Fit between Research design domains and Research conduct domain, Fit between Research conduct domains and Researchers, Fit between Research design domains and Stakeholders, Fit between Research conduct domains and Stakeholders, Fit between Research conduct domains and Context, Fit between Research design domains and Research aim/questions, Fit between Research conduct domains and Research aim/questions, Fit between Research design domains and Context) | No or very minor concerns | Minor concerns:  8 out of 26 papers are indirectly relevant to the review aim. Contributing papers represented 19 countries in five regions, AFRO (Malawi, Tanzania, Ghana, Benin, Burkina Faso), EMRO (Morocco, Iran), EURO, (Turkey, Ireland, The Netherlands, Belgium, Israel, UK, Switzerland ), PAHO (USA, Canada), WPRO (Australia, China, New Zealand), where 10 are high income, 2 are upper middle income, 6 are lower middle income and 1 is low income. Perspectives came from midwives, nurses, nurse-midwives, and doctors. | No or very minor concerns | Moderate confidence | No or very minor concerns on coherence, no or very minor concerns on relevance, no or very minor concerns on adequacy, moderate concerns on methodological limitations (fit between research design domains and researchers, fit between research design domains and research conduct domain, fit between research conduct domains and researchers, fit between research design domains and stakeholders, fit between research conduct domains and stakeholders, fit between research conduct domains and context, fit between research design domains and research aim/questions, fit between research conduct domains and research aim/questions, fit between research design domains and context) |  |  |  |  |  |  |  |
| **Theme 5: Support systems for well-being** | | | | | | | | |  |  |  |  |  |  | **Support systems for well-being** |
| 15 | **Receiving reliable peer support.** Support from colleagues and within the professional community was a crucial coping strategy for addressing workplace stress, exhaustion, and burnout among health workers. When health workers had colleagues with whom they could share their feelings and experiences, seek advice, receive reassurance, or even a comforting hug, it helped alleviate feelings of isolation and ensured a sense of safety in their practice. | (36, 37, 43-45, 48, 51-55, 61, 62, 66, 67, 69-75, 78, 83) | Moderate concerns:  2 papers with no or minor concerns, 6 with minor concerns (Fit between Research design domains and Researchers, Fit between Research conduct domains and Researchers), 9 with moderate concerns (Fit between Research design domains and Researchers, Fit between Research design domains and Context, Fit between Research conduct domains and Researchers, Fit between Research conduct domains and Context), 7 with serious concerns (Fit between Research design domains and Researchers, Fit between Research design domains and Research conduct domain, Fit between Research conduct domains and Researchers, Fit between Research design domains and Stakeholders, Fit between Research conduct domains and Stakeholders, Fit between Research design domains and Context, Fit between Research conduct domains and Context, Fit between Research conduct domains and Research aim/questions) | No or very minor concerns | Minor concerns:  6 out of 24 papers are indirectly relevant to review's aim. Contributing papers represented 15 countries in four regions, EMRO (Morocco, Iran ), EURO (Norway, Turkey, Ireland, The Netherlands, Belgium, Israel, UK, Sweden), PAHO (USA, Canada), WPRO (New Zealand, Australia, China), where 11 are high income, 2 are upper middle income, and 2 are lower middle income. Perspectives came from midwives, obstetricians, and nurses. | No or very minor concerns | Moderate confidence | No or very minor concerns on coherence, no or very minor concerns on adequacy, minor concerns on relevance (24 papers represented 11 high income countries), moderate concerns on methodological limitations (fit between research design domains and researchers, fit between research design domains and research conduct domain, fit between research conduct domains and researchers, fit between research design domains and stakeholders, fit between research conduct domains and stakeholders, fit between research design domains and context, fit between research conduct domains and context, fit between research conduct domains and research aim/questions) |  |  |  |  |  |  |  |
| 16 | **Accessing adequate mentoring, supervision and leadership.** Health workers described that adequate mentoring and supervision, along with strong leadership, were crucial for creating a healthier work environment, including managing difficult workplace situations and reducing workplace stress. | (35-37, 40, 41, 43, 44, 48-51, 53-56, 58, 59, 61, 63, 66, 70-74, 76, 77, 83, 84) | Moderate concerns:  1 papers with no or minor concerns, 12 with minor concerns (Fit between Research design domains and Researchers, Fit between Research conduct domains and Researchers, Fit between Research design domains and Context, Fit between Research conduct domains and Context), 8 with moderate concerns (Fit between Research design domains and Researchers, Fit between Research design domains and Context, Fit between Research conduct domains and Researchers, Fit between Research conduct domains and Context, Fit between Research design domains and Stakeholders), 8 with serious concerns (Fit between Research design domains and Researchers. Fit between Research conduct domains and Researchers, Fit between Research design domains and Research conduct domain, Fit between Research conduct domains and Research aim/questions, Fit between Research design domains and Stakeholders, Fit between Research conduct domains and Stakeholders, Fit between Research conduct domains and Context, Fit between Research design domains and Research aim/questions. | No or very minor concerns | Minor concerns:  11 out of 29 papers are indirectly relevant to the review aim. Contributing papers represented 16 countries in 5 regions, AFRO (Ghana, South Africa), EMRO (Morocco, Iran), EURO (Norway, Turkey, Ireland, UK, Israel, Sweden), PAHO (USA,), WPRO (New Zealand, Australia), where 9 are high income, 2 are upper middle income, 4 are lower middle income and 1 is low income. Perspectives came from midwives, obstetricians, nurse-midwives and nurses. | Minor concerns: 29 out of 51 papers contributed to the review finding (21 moderately thick and 8 thin data) | Moderate confidence | No or very minor concerns on coherence, minor concerns on relevance (11 papers with indirectly relevant aim), minor concerns on adequacy (29 of 51 papers contributed; 21 thick and 8 thin data ), moderate concerns on methodological limitations (fit between research design domains and researchers. fit between research conduct domains and researchers, fit between research design domains and research conduct domain, fit between research conduct domains and research aim/questions, fit between research design domains and stakeholders, fit between research conduct domains and stakeholders, fit between research conduct domains and context, fit between research design domains and research aim/questions |  |  |  |  |  |  |  |
| 17 | **Requiring institutional support.** Health workers required institutional support from their workplace, including mental health, emotional support and guidance. Many workplaces failed to provide this support, and when available, the support often focused on investigating the clinical aspects of the event or criticising health workers, rather than addressing the emotional needs of health workers. | (34, 36, 37, 39, 43-45, 48, 51, 53, 54, 56, 58, 59, 62, 65, 66, 71-73, 77, 82, 83) | Moderate concerns:  3 papers with no or minor concerns, 7 with minor concerns (Fit between Research design domains and Researchers, Fit between Research conduct domains and Researchers), 7 with moderate concerns (Fit between Research design domains and Researchers, Fit between Research design domains and Context, Fit between Research conduct domains and Researchers, Fit between Research conduct domains and Context), 7 with serious concerns (Fit between Research design domains and Researchers, Fit between Research conduct domains and Researchers, Fit between Research design domains and Research conduct domain, Fit between Research conduct domains and Research aim/questions, Fit between Research design domains and Stakeholders, Fit between Research conduct domains and Stakeholders, Fit between Research design domains and Research aim/questions, Fit between Research conduct domains and Context) | No or very minor concerns | No or very minor concerns: | Minor concerns: 24 out of 51 papers contributed to the review finding (16 moderately thick and 8 thin data) | Moderate confidence | No or very minor concerns on coherence, no or very minor concerns on relevance, minor concerns on adequacy (24 of 51 papers contributed; 16 moderately thick and 8 thin data ), moderate concerns on methodological limitations (fit between research design domains and researchers, fit between research conduct domains and researchers, fit between research design domains and research conduct domain, fit between research conduct domains and research aim/questions, fit between research design domains and stakeholders, fit between research conduct domains and stakeholders, fit between research design domains and research aim/questions, fit between research conduct domains and context) |  |  |  |  |  |  |  |
| 18 | **Building social network support.** Family and friends played a dual role in supporting health workers. Some health workers perceived that family and friends served as valuable support systems when facing workplace challenges, while others avoided or found limited support in their family and friends. | (36, 37, 43, 44, 49, 52-55, 58, 62, 66, 67, 72, 77, 78) | Moderate concerns:  1 paper with no or minor concerns, 5 with minor concerns (Fit between Research design domains and Researchers), 3 with moderate concerns (Fit between Research design domains and Researchers, Fit between Research design domains and Context, Fit between Research conduct domains and Researchers, Fit between Research conduct domains and Context), 7 with serious concerns (Fit between Research design domains and Researchers, Fit between Research design domains and Research conduct domain, Fit between Research conduct domains and Research aim/questions, Fit between Research conduct domains and Researchers, Fit between Research design domains and Stakeholders, Fit between Research conduct domains and Stakeholders, Fit between Research conduct domains and Context, Fit between Research design domains and Context) | No or very minor concerns | Minor concerns:  5 out of 16 papers are indirectly relevant to the review aim. Contributing papers represented 14 countries in 5 regions, AFRO (South Africa), EMRO (Morocco, Iran), EURO (Norway, Turkey, Ireland, The Netherlands, Belgium, Israel, UK), PAHO (Canada, USA), WPRO (New Zealand, China), where 9 are high income, 3 are upper middle income, and 2 are lower middle income. Perspectives came from midwives, obstetricians, and nurses. | Moderate concerns:  16 out of 51 papers contributed to the review finding (6 moderate and 10 thin data) | Moderate confidence | No or very minor concerns on coherence, minor concerns on relevance (majority of papers represented countries in EURO region), moderate concerns on adequacy (16 of 51 papers contributed; 6 moderate and 10 thin data), moderate concerns on methodological limitations (fit between research design domains and researchers, fit between research design domains and research conduct domain, fit between research conduct domains and research aim/questions, fit between research conduct domains and researchers, fit between research design domains and stakeholders, fit between research conduct domains and stakeholders, fit between research conduct domains and context, fit between research design domains and context) |  |  |  |  |  |  |  |
