# Supplementary material for: Well-being of health workers providing maternal and newborn care: A qualitative evidence synthesis
Source: PLOS Glob Public Health. 2026 Feb 11;6(2):e0005522. doi: 10.1371/journal.pgph.0005522 (PMC12893595; doi:10.1371/journal.pgph.0005522)
Supplement: S8 Appendix — (DOCX) [file pgph.0005522.s008.docx]

## S8 Appendix. Well-being domains for health workers providing maternal and newborn health care


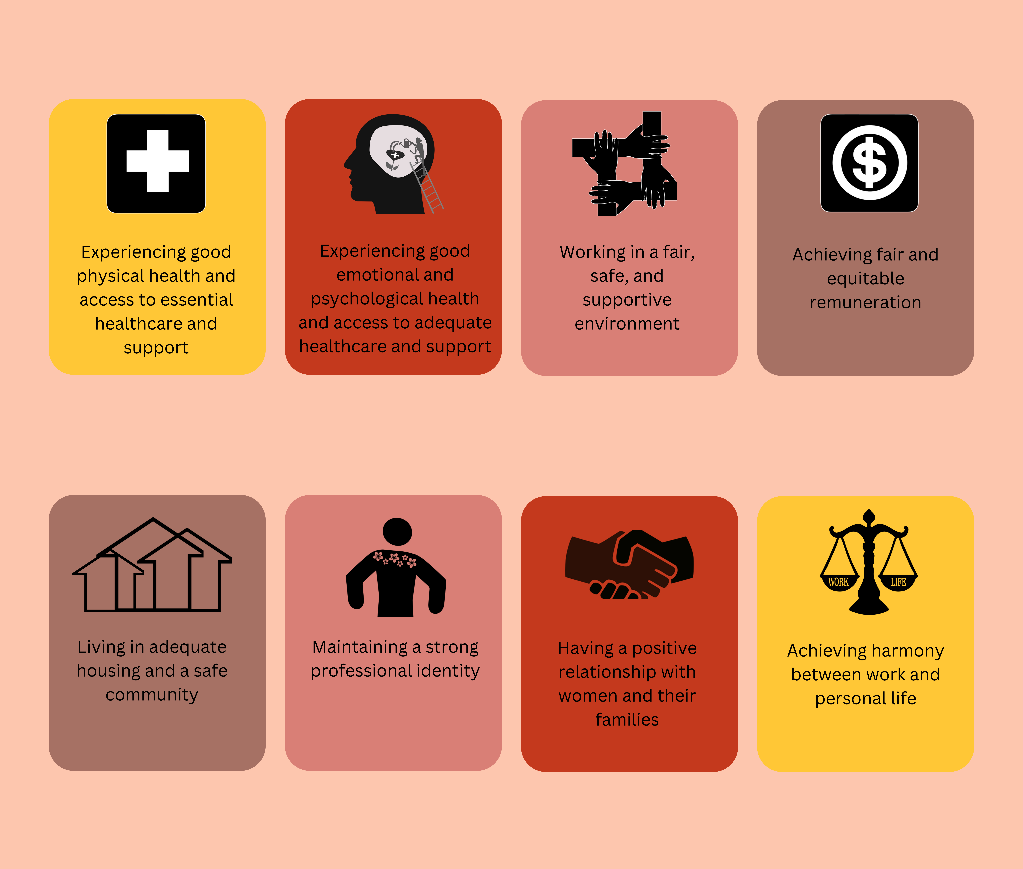


Figure 3. Health workers' well-being domains

**Domain 1:**  *Experiencing good physical health and access to essential healthcare and support.* Maintaining good physical health, including being free from disease and injuries and being able to perform their work without restrictions, and ensuring adequate access and support to health care are crucial for health workers to provide high-quality care.

**Domain 2:** *Experiencing good emotional and psychological health and access to adequate healthcare and support*. Having have good emotional and physiological health, including being able to manage and express their positive and negative emotions, and accessing adequate mental health services and support, which can include both formal support (e.g., professional support, institutional-based support) and informal support (e.g., support from their social networks) are crucial for health workers to support them to provide high-quality care.

**Domain 3:** *Working in a fair, safe, and supportive environment.* A fair, safe, and supportive environment involves a healthy workplace culture, reliable support from colleagues, good workload management, effective mentoring, supervision, and leadership, opportunities to enhance their knowledge and skills, support for their career progression, and adequate infrastructure and policies to prevent any safety issues and injuries.

**Domain 4:** *Achieving fair and equitable remuneration.* Fair and equitable remuneration for health workers is essential, as it reflects their value, enhances job satisfaction and security, and ultimately improves their well-being and motivation in their roles.

**Domain 5:** *Living in adequate housing and a safe community.* Access to secure, stable, and high-quality housing with essential amenities, such as clean water, electricity, and education facilities for their children, is crucial for health workers' well-being, particularly in rural areas, significantly impacting their capacity to provide care.

**Domain 6:** *Maintaining a strong professional identity.* A strong professional identity, built on the autonomy to perform their duties, a connection to their professional expertise and peer groups, and a sense of passion, compassion, and responsibility, is crucial for health workers’ resilience and confidence.

**Domain 7:** *Having a positive relationship with women and their families.* Health workers value their relationships with women and their families, which can lead to positive emotional experiences for health workers and help alleviate emotional burden.

**Domain 8:** *Achieving harmony between work and personal life.* Work-life harmony, supported by strong support systems at work and home and the ability to practice spirituality, is essential for health workers to manage stress, prevent burnout, and build resilience.
